# Chitosan/Alginate Polyelectrolyte Magnetic Gel Nanoarchitectonics with Tunable Mechanical Properties for Magnetic Hyperthermia and Sustained Release of 5‑Fluorouracil

**DOI:** 10.1021/acsami.5c15863

**Published:** 2025-10-28

**Authors:** Sérgio R. S. Veloso, Margarita Vázquez-González, Mariana O. Ribeiro, Loic Hilliou, Carlos O. Amorim, Vítor S. Amaral, Miguel A. Correa-Duarte, Elisabete M. S. Castanheira

**Affiliations:** † Physics Centre of Minho and Porto Universities (CF-UM-UP), 56059 University of Minho, Campus de Gualtar, 4710-057 Braga, Portugal; ‡ LaPMET Associate Laboratory, 56059 University of Minho, Campus de Gualtar, 4710-057 Braga, Portugal; § Centro de Investigación en Nanomateriais e Biomedicina (CINBIO), 16784 Universidad de Vigo, 36310 Vigo, Spain; ∥ Institute for Polymers and Composites, Department of Polymer Engineering, 56059 University of Minho, Campus de Azurém, Guimarães, 4800-058, Portugal; ⊥ Physics Department and i3N, University of Aveiro, Campus de Santiago, Aveiro 3810-193, Portugal; # Physics Department and CICECO, University of Aveiro, Campus de Santiago, Aveiro 3810-193, Portugal

**Keywords:** chitosan, alginate, magnetic gels, magnetic hyperthermia, drug delivery

## Abstract

Chitosan-based hydrogels hold promise as drug delivery systems for cancer therapy, but the poor mechanical properties often limit the biological application, requiring chemical cross-linking to improve sustained drug release. Besides, the addition of stimulus-responsiveness to chitosan requires chemical modifications that can further affect the gel properties. To overcome these challenges, in this work, a novel chitosan/alginate polyelectrolyte magnetic gel with tunable mechanical properties is developed by pH-triggered self-assembly. The gels could be prepared by a slow/fast pH decrease and blended with magnetic nanoparticles. Manganese-doped ferrite nanoparticles (∼10 nm) with suitable magnetic properties (>70 Am^2^/kg) and high magnetic hyperthermia heating efficiency (ILP > 3 nHm^2^/kg) were synthesized via an amino acid-assisted oxidative hydrothermal method. The nanoparticles and self-assembly conditions of the polyelectrolyte complex enabled the tuning of the gels’ properties, a fast gelation, and suitable mechanical properties for drug delivery. Notably, gels with a large storage modulus (up to 10 kPa) could be prepared at a low polymer concentration (≤2 wt %). The magnetic gels enabled the sustained release of a hydrophilic chemotherapeutic drug model, 5-fluorouracil (5-FU), under mimetic physiological conditions, outperforming the hydrogels. Moreover, the drug release kinetics was synergistically enhanced under the combined effect of acidic conditions and magnetic hyperthermia. Hence, the developed self-assembled chitosan/alginate magnetic gel showed promising multifunctionality, combining tunable mechanical properties, magnetic hyperthermia capability, and sustained drug release. These features highlight the self-assembled chitosan/alginate magnetic gels as promising and versatile materials for localized and controlled drug delivery.

## Graphical abstract

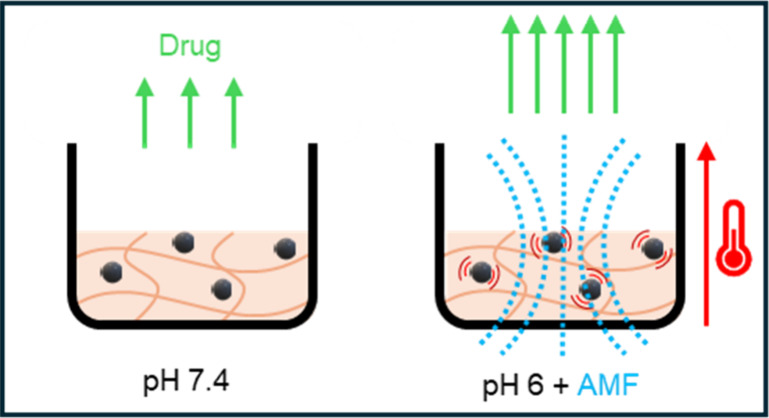

## 1 Introduction

Chitosan is a natural polycationic polymer obtained by the deacetylation of chitin (second most abundant organic compound on Earth), consisting of β-(1→4)-linked d-glucosamine and *N*-acetyl-d-glucosamine monomer units.^1,2^ This biopolymer is highly advantageous for several biomedical applications owing to the biocompatibility, biodegradability, and wound healing ability, as well as mucoadhesive and antibacterial properties.^1–4^ Besides, it has shown promising results in the development of drug delivery systems for a wide variety of drugs.^5^ However, the poor water solubility,^3^ incapability to form hydrogels,^1^ and/or the weak mechanical toughness of chitosan-based gels^6^ can limit the applicability in certain fields, including drug delivery, requiring chemical modifications and/or chemical cross-linking, thus sacrificing the low toxicity and biological activity. Instead, the combination of chitosan with oppositely charged polymers, forming polyelectrolyte complexes (PECs), has provided a suitable strategy to tune the gel’s properties while retaining the chitosan’s unique advantages.^1,2,6^ Among the anionic polymers, alginate, a natural biopolymer consisting of (1,4)-β-d-mannuronate and α-l-guluronate, is reported to enable the formation of chitosan-based PEC gels at physiological pH.^4,7,8^ These gels can be tuned by adjusting pH, concentration, and ratio of chitosan/alginate, charge density, ionic strength, and temperature. Yet, chitosan/alginate gels assessed for drug delivery mostly consist of chemically modified or cross-linked chitosan,^5^ while the physical chitosan/alginate gels have been poorly explored despite the suitable mechanical properties. Besides, the hydrophilic behavior of chitosan makes the controlled release of hydrophilic drugs a challenging task, thus often requiring chemical modification.

The combination of gels with magnetic nanoparticles, into magnetic gels,^9,10^ is a promising approach to improve the sustained release of hydrophilic drugs without requiring chemical modification of the gel components.^11,12^ Besides, the magnetic hyperthermia achieved by an externally applied alternating magnetic field can synergistically enhance the drug’s distribution, cells’ sensitivity to chemotherapeutic drugs, and therefore efficacy.^13,14^ Thus, well-dispersed and stable magnetic nanoparticles with high heating generation efficacy are required.^12^ However, reported chitosan-based magnetic gels commonly include magnetite or cobalt-doped ferrite nanoparticles,^12^ while manganese-doped ferrites can show improved biocompatibility and magnetic properties for biomedical applications.^15,16^

In drug delivery, chitosan-based magnetic gels showed promising results for the delivery of 5-fluorouracil (5-FU),^14,17,18^ doxorubicin,^19^ dexamethasone,^20^ and riboflavin.^21^ Particularly, 5-FU is a major anticancer therapeutic, which is described to inhibit thymidylate synthase, depleting deoxythymidine triphosphate required for DNA replication and repair, and thus inducing cancer cell death.^22^ It is used for various malignant tumors, including breast, pancreatic, skin, stomach, esophageal, and head and neck cancers,^23^ but it has several limitations, such as short half-life, high cytotoxicity, and low bioavailability.^24^

Hereby, in this work, a self-assembled chitosan/alginate PEC magnetic gel with tunable mechanical properties was developed to improve the sustained and controlled release of 5-FU through combination with magnetic nanoparticles. In this way, manganese-doped ferrites with improved magnetic properties were prepared by oxidative hydrothermal synthesis.^15^ For this, amino acids (AA = phenylalanine, alanine, glycine, asparagine, cysteine, and glutamate) were proposed here as in situ synthesis ligands. Additionally, the self-assembly conditions of chitosan/alginate gels were explored to assess their effect on the mechanical properties. In Scheme 1 is included a graphical summary of the synthesis of magnetic nanoparticles and development of the magnetic gel. A slightly alkaline chitosan solution was prepared using NaHCO_3_,^2–4,7,25^ mixed with alginate, and combined with the ferrite nanoparticles (and 5-FU) to obtain the magnetic gels through addition of phosphate buffer pH 7.4 (pH-switch method) or slow pH decrease using GdL (GdL method). The drug-loaded magnetic gels were compared with the neat hydrogels. Importantly, the magnetic gels displayed an improved sustained release of 5-FU, which could be enhanced through magnetic hyperthermia. Hence, the chitosan/alginate PEC magnetic gel described herein holds promise for drug delivery.

**Scheme 1:**
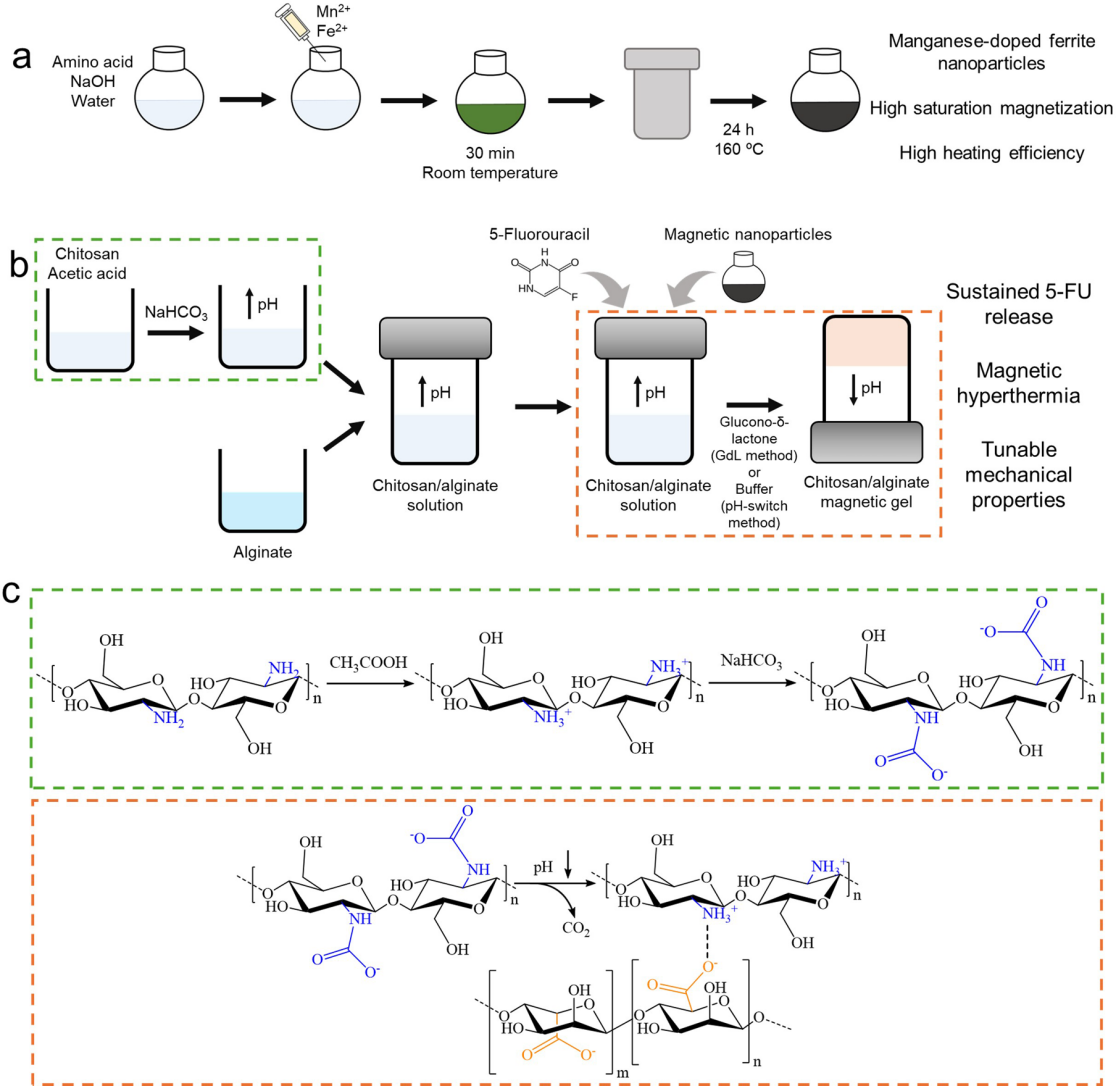
Schematic Summary of Chitosan/Alginate Magnetic Gels^a^

a (**a**) Manganese-doped ferrite nanoparticles were synthesized through amino acid-assisted oxidative hydrothermal synthesis. The assessed amino acids (AA) were phenylalanine (Phe), alanine (Ala), glycine (Gly), asparagine (Asn), cysteine (Cys), and glutamate (Glu). (**b**) A basic solution of chitosan was prepared using NaHCO_3_, which was posteriorly mixed with alginate, magnetic nanoparticles, and 5-fluorouracil. The addition of glucono-δ-lactone (GdL, GdL method) or phosphate buffer (pH 7.4, pH-switch method) induced a pH decrease and consequent gelation. The magnetic gel enabled the sustained release of 5-fluorouracil (5-FU), which could be enhanced through magnetic hyperthermia. (**c**) Scheme of the reaction of chitosan with sodium bicarbonate, regeneration of primary amines upon pH decrease, and interaction with sodium alginate.^4^

## 2 Results and Discussion

### 2.1 Development of Amino Acid-Functionalized Manganese-Doped Ferrite Nanoparticles

FTIR measurements (Figure 1a, Figure S1) confirmed the surface functionalization with AA as suggested by the peaks near ∼1620 cm^–1^ and 1400 cm^–1^, which are assigned to the asymmetric (ν_as_(COO−)) and symmetric (ν_s_(COO−)) stretching of the carboxyl group, respectively. These peaks are commonly associated with bridging or ionic coordination between the AA carboxyl anions and the nanoparticles’ surface cations.^26^ The spectra also display the ν­(CH) stretching in the range 2800–3000 cm^–1^ overlapping the AA’s ν­(NH) and nanoparticles’ ν­(OH) vibrations in the range 3200–3400 cm^–1^. In line with other manganese-doped ferrites,^27^ the nanoparticles’ ν­(MO) lattice vibration is observed at ∼526 cm^–1^, except for the cysteine-functionalized nanoparticles, in which the strong peaks at ∼516 cm^–1^ and ∼435 cm^–1^ suggest the presence of hematite.^28^

**Figure 1:**
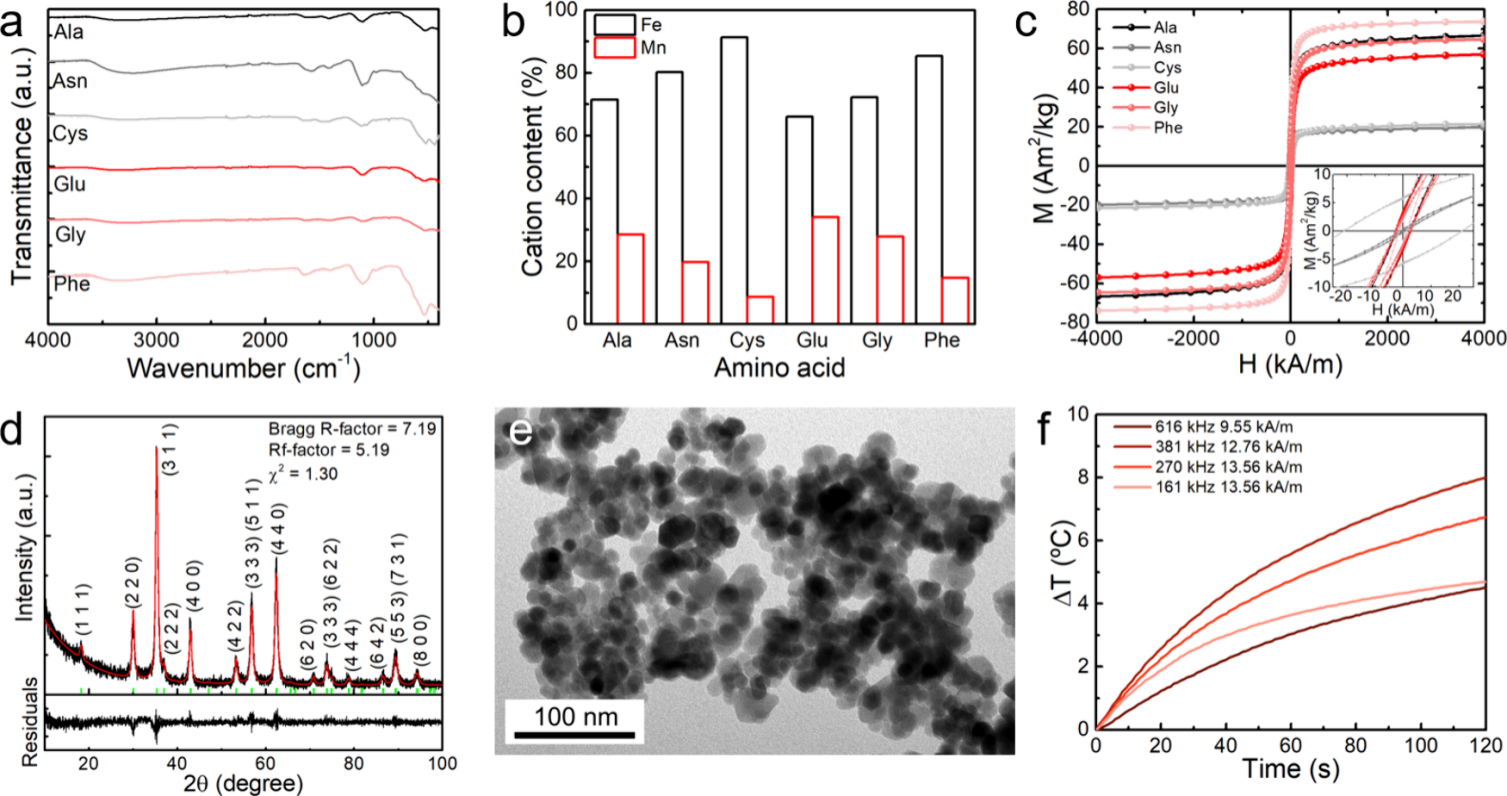
Comparison of the (**a**) FTIR spectra, (**b**) cation composition obtained through ICP-OES, and (**c**) magnetization dependence on the applied magnetic field of the amino acid-functionalized manganese-doped ferrite nanoparticles. Inset: enlargement of the loops in the low-field region. (**d**) XRD diffraction pattern and fitted Rietveld refinement pattern, (**e**) TEM image, and (**f**) temperature variation of Phe-functionalized nanoparticles under different alternating magnetic field conditions.

Interestingly, the amino acids were found to affect the cation composition (Figure 1b, Table S1), in which Ala, Gly, and Glu provided nanoparticles with the closest experimental Mn:Fe content to the theoretical ratio (1:2). The synthesis with amino acids is described to affect the crystal phase,^29^ size, and shape of the iron oxide nanoparticles,^30^ while carboxylate and amine ligands are also known to influence the cation distribution.^31^ In this way, the AAs were anticipated to affect the magnetic properties, in which the highest saturation magnetization was obtained for Phe (73 A.m^2^/kg), outperforming other manganese-doped ferrites,^32^ and ferrites functionalized with amino acids,^33^ while Asn (18 Am^2^/kg) and Cys (20 Am^2^/kg) afforded the nanoparticles with the lowest values (Figure 1c). Also, the nanoparticles displayed mostly superparamagnetic behavior at 300 K, including low values of coercivity and remanence magnetization, and squareness < 0.05 (Table S2), except for the particles functionalized with Cys. Importantly, the saturation magnetization was found to increase with iron content, rendering Glu-functionalized nanoparticles the largest iron-normalized saturation magnetization (201.4 Am^2^/kg_Fe_), which is near the pure bulk iron (218 Am^2^/kg).^34^ However, a low magnetization was obtained for Cys- and Asn-functionalized nanoparticles despite a large iron content, which can be associated with the amorphous-like structures. Particularly, Cys produced cluster-like nanoparticles that are commonly characterized by a larger coercivity.^15^ The remaining AAs afforded nearly monodisperse spherical particles within the size range of 9–16 nm (Figures S2 and S3) and with suitable magnetic properties.

Following these results, and considering the dependence of the intrinsic loss power on saturation magnetization (ILP ∝ *M*_s_^2^),^35^ the Phe-functionalized nanoparticles were analyzed by XRD (Figure 1d) and employed in the development of magnetic gels. The nanoparticles displayed a single phase of cubic spinel (*Fd*3*m* space group) with a crystallite size of 11.4 nm, slightly smaller than that obtained from TEM (Figure 1e, 15.6 ± 3.5 nm), and a lattice parameter (*a* = 8.415(9) Å) near the values reported for MnFe_2_O_4_ nanoparticles with similar size.^36^ The crystallinity of other nanoparticles was also confirmed (Figure S4). Regarding the heating performance (Figure 1f), the notably high ILP > 1.5 nHm^2^/kg (5 mg/mL) for several magnetic field conditions overcomes manganese ferrite nanoparticles obtained by citrate-assisted oxidative hydrothermal synthesis^15^ and other synthesis methods that afforded a similar particle size.^37–40^ The heating performance was also assessed for the remaining nanoparticles (Figure S5 and Table S3), in which the Phe- and Cys-prepared nanoparticles displayed the highest and lowest ILP values, respectively, in line with the magnetization results.

### 2.2 Development and Characterization of Hydrogels

Chitosan/alginate gels could be prepared by initially mixing alginate with a basic chitosan solution, followed by acidification with glucono-δ-lactone (GdL). Considering chitosan is a cationic polysaccharide (p*K*_a_ = 6.5) insoluble in neutral and basic pH, its dissolution required the modification of amines to carbamate through addition of sodium hydrogen carbonate in acetic acid solution.^2,4,25^ Gradual acidification by GdL leads to cleavage of the carbamate groups, enabling electrostatic interaction of the chitosan amines with the oppositely charged carboxylic group. As the pH decreased from ∼8.1 to values below 7.0, the zeta potential progressively became less negative with the incremental content of GdL (Figure S6). The resulting gels were found to comprise a rough surface-like morphology (Figure 2a) with pores smaller than 200 nm, as measured from TEM images (Figure S7), and a critical gelation concentration (CGC) of 1 wt % total polymer (chitosan/alginate 1:1 molar ratio), requiring at least 0.5 wt % of GdL (final pH ∼ 7.1), as displayed in the assembly conditions diagram (Figure 2b).

**Figure 2:**
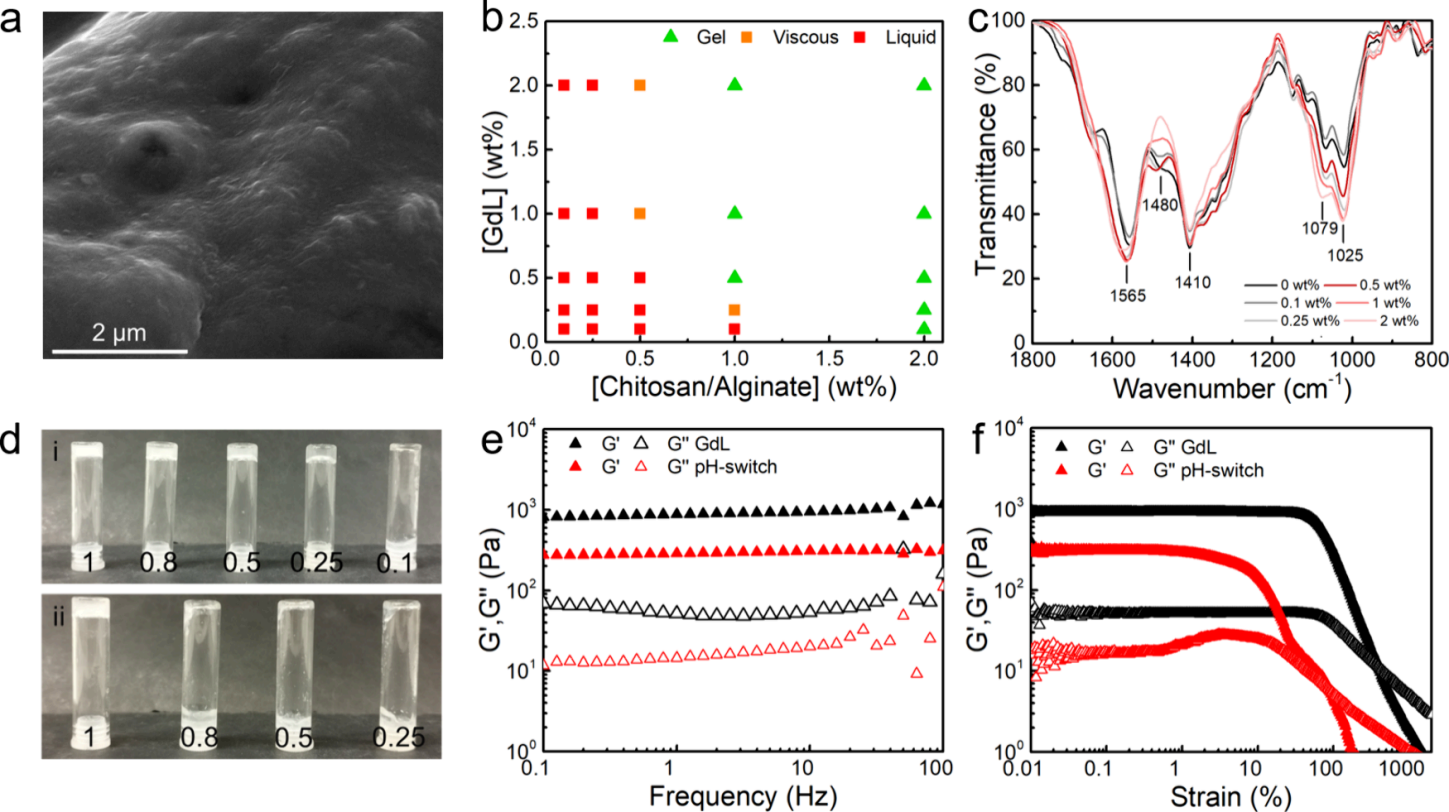
(**a**) SEM image of the chitosan/alginate hydrogel. (**b**) Phase transition diagram of the chitosan/alginate polyelectrolyte complex (PEC) prepared through addition of GdL to a basic pH PEC solution. (**c**) FTIR spectra of chitosan/alginate PEC (1 wt %) prepared with different GdL concentrations. (**d**) Vial inversion test on chitosan/alginate PEC (1 wt %) triggered by (i) GdL (varying GdL concentration from 0.1 to 1 wt %) and (ii) pH-switch (varying polymer concentration from 0.25 to 1 wt %) method. (**e**) Frequency and (**f**) strain sweep of the gels prepared by the GdL and pH-switch methods.

FTIR spectroscopy further confirmed the formation of chitosan/alginate polyelectrolyte complexes (PECs), showing the characteristic broad bands near 1565 cm^–1^ and 1410 cm^–1^ (Figure 2c),^4^ which include overlapping bands from the chitosan’s amine and alginate’s carboxylate. Incremental GdL content led to a red-shift of ν­(C=O) at 1700 cm^–1^ and a blue-shift of δ­(NH) at 1560 cm^–1^ (Figure S8), suggesting the electrostatic interaction between the amine and carboxylate groups. Importantly, this effect is noticeable at low GdL concentration (<0.5 wt %), despite the negligible change in zeta potential. Additional shifts of the peaks near 1480 cm^–1^, 1410 cm^–1^, 1025 cm^–1^, and 1079 cm^–1^ further suggest the PEC formation. In this way, considering the gelation at low GdL concentration with pH ∼ 7, gels could also be formed with the addition of buffer pH 7.4 (pH switch method) with a CGC of 1 wt % (Figure 2d).

Rheological assays were carried out to understand the effect of the preparation conditions on the mechanical properties of the chitosan/alginate gels. Notably, gel-like properties were obtained from the onset of time sweep measurements (Figure S9), but with a faster gelation kinetics by pH switch than GdL, which can be due to the latter’s slower hydrolysis limiting the rate of pH decrease and, consequently, the PEC formation. Despite differences in storage (*G*′) and loss (*G*″) moduli (Figure 2e), both gels displayed a nearly frequency-independent *G*′ 10× larger than *G*″, confirming the solid-like nature. Importantly, the gels fall in the range of native tissues’ and organs’ elastic modulus^41^ and could achieve a larger range of storage modulus and yield point than other chitosan/alginate gels^4,7^ and chitosan-based gels prepared by chemical cross-linking (Table S4).^42–45^ Besides, the here-developed gels could be prepared at a low polymer concentration and at neutral pH, achieving *G*′ ∼ 883 and 288 Pa at 1 wt % using GdL and a pH switch, respectively. By comparison, chitosan-based gels prepared by self-assembly using HCl or genipin typically yield *G*′ < 100 Pa (2 wt % of polymer), which are applied in drug delivery or wound healing.^7,43^ Chemically cross-linked gels, such as chitosan/methacrylate gelatin gels with *G*′ ≈ 300 Pa (at 2.5 wt %)^45^ and chitosan/collagen gels formed with various cross-linkers with *G*′ ranging from 150 to 529 Pa (at 1.5 wt %) have been applied in tissue engineering.^42^ In this way, the developed polyelectrolyte chitosan/alginate gels can achieve a broader range of tunable mechanical properties at lower polymer concentrations, making them potential candidates to be assessed for several biomedical applications.

Regarding the strain sweeps (Figure 2f), a different behavior was obtained for both gels. The GdL method led to a larger *G*′, linear viscoelastic region (LVR), and yield point (crossover of *G*′ and *G*″), as well as a strain thinning behavior. This behavior is characteristic of gels in which the network chains are easily lost or the dangling chain segments are unable to rejoin the network structure as the network chains/microstructures align along the flow direction.^46^ Oppositely, the pH-switch gels withstood less deformation and displayed a weak strain overshoot. The fast gelation of self-assembled gels is described to result in weaker gels due to the formation of local network inhomogeneities.^47^ Besides, the weak strain overshoot is commonly associated with the rearrangement of network microstructures.^46,48^

Increasing the amount of total polymer and GdL was found to enhance the gelation kinetics, yield point, and *G*′ (Figures S10 and S11). From classical elasticity theory, this could be a result of a larger entanglement or cross-linking density. However, above a critical concentration, the decreasing *G*′ and yield points suggested that the mechanical properties are influenced by the chitosan and alginate pH-dependent degree of ionization. Considering the PEC gel is formed due to the electrostatic interaction between the oppositely charged polymers, an excess of positive or negative charges can lower the degree of complexation, which reduces the physical cross-linking.^49^ Furthermore, these changes were accompanied by the modification of the rheological profile, thus indicating that the network structure is also affected by the complexation changes between chitosan and alginate.

### 2.3 Development and Characterization of Magnetogels

Magnetic gels, which comprise chitosan/alginate gels loaded with magnetic nanoparticles, could support their own weight up to 1 mg/mL of nanoparticles (Figure 3a). The homogeneity was confirmed by SEM and TEM (Figures 3b and 3c), in which composites are distributed across the hydrogel matrix, forming some clusters, as reported in other self-assembled magnetic gels. In Figure 3c, the nanoparticles are observed to occupy the nanoscale pores of the gel matrix. Additional images are included in Figure S12.

**Figure 3:**
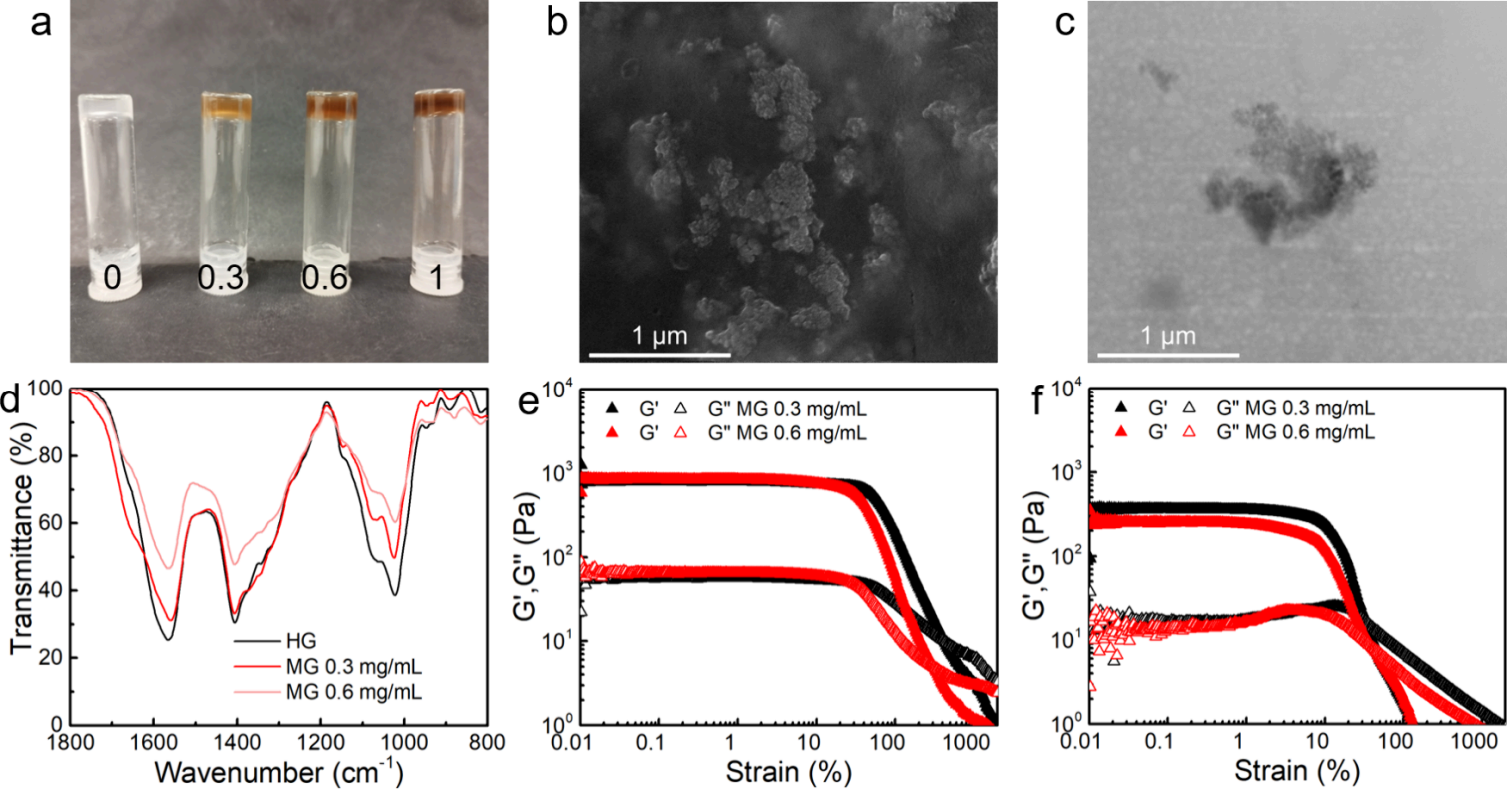
(**a**) Vial inversion test on chitosan/alginate gels (1 wt %) triggered by GdL and loaded with 0, 0.3, 0.6, and 1 mg/mL of Phe-functionalized nanoparticles. (**b**) SEM and (**c**) TEM images of the magnetic gels. (**d**) FTIR spectra of chitosan/alginate hydrogel (HG) and magnetic gel (MG) prepared with variable content of magnetic nanoparticles. Strain sweeps of magnetic gels prepared by the (**e**) GdL and (**f**) pH-switch method and loaded with 0.3 or 0.6 mg/mL of magnetic nanoparticles.

FTIR spectra (Figure 3d) of the magnetic gels displayed slight shifts in peaks near 1025 cm^–1^, 1565 cm^–1^, and 1700 cm^–1^, suggesting that the nanoparticles interact with the gel network structure. However, the effect of the nanoparticles on the mechanical properties was found to depend on the self-assembly pathway (Figures 3e and 3f). For instance, the *G*′ of magnetic gels prepared by the GdL method remained similar to that of the hydrogel, while a slight decrease was obtained for the pH-switch gels. This effect was also described for other classes of physical gels.^13,50,51^ The gelation kinetics (Figure S13) and rheological profiles remained similar across the assessed nanoparticle concentration but with a subtle decrease of the yield point. In this sense, depending on the self-assembly pathway, the nanoparticles can interact with the gel structure and modify the cross-linking density. As a result, the combination with composites provides an additional degree of tunability of chitosan/alginate gels’ mechanical properties. Importantly, the developed magnetic gels could also attain a comparable gelation kinetics and higher *G*′ than reported chemical or physical cross-linked chitosan-based magnetic gels used for drug delivery and tissue engineering (Table S4).^52–55^ For instance, the developed gels (*G*′ ∼ 750 Pa for GdL and *G*′ ∼ 345 Pa for pH switch) fall within a range of *G*′ values reported for chitosan/dialdehyde sodium alginate gels (*G*′ ∼ 1800–8900 Pa)^53^ and chitosan/carboxymethyl chitosan gels prepared with NaCl (*G*′ ∼ 400 Pa),^54^ both of which are applied in drug delivery. Additionally, the magnetic polyelectrolyte gels’ *G*′ values are also higher than those of magnetic chitosan gels prepared with β-glycerophosphate (*G*′ ∼ 200 Pa), which have been applied for myocardial infarction treatment.^55^ Despite that direct comparisons remain challenging since polymer concentrations are often not specified in the literature, the magnetic polyelectrolyte gels are able to outperform other formulations that require chemical cross-linking and attain mechanical properties of interest for different biomedical applications, including drug delivery.

### 2.4 Magnetic Heating Performance of Magnetic Gels

Before controlled drug delivery was studied, the heating efficiency of the nanoparticles in the gel was assessed. Magnetic gels with 0.3 mg/mL (Figure 4a) of nanoparticles could produce a heating of ∼5 °C in 30 min, achieving ILP values larger than 3 nHm^2^/kg.

**Figure 4:**
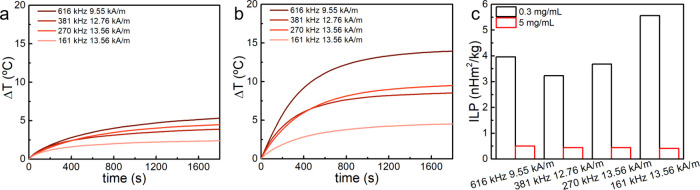
Temperature variation in magnetic gels with (**a**) 0.3 and (**b**) 5 mg/mL of magnetic nanoparticles under different magnetic field strength and frequencies and (**c**) the respective intrinsic loss power (ILP) values.

For 5 mg/mL of nanoparticles, the gels achieved a temperature variation up to ∼14 °C (Figure 4b), but the ILP values were lower than in solution and also compared to a lower particle concentration (Figure 4c, Table S5). The quenching of the heating efficiency of magnetic nanoparticles in gels has been described elsewhere^13,50,51^ and can be associated with the loss of Brownian motion when immobilized in the gel matrix and the deleterious effect of dipolar interaction in particle aggregates.^50,56^ Additionally, larger ILP values were obtained by increasing the frequency and decreasing the amplitude of the AMF (Figure S14), as described elsewhere.^57^

### 2.5 Drug Release Assays

Drug release assays were carried out for both the gels prepared by the GdL and pH-switch methods with the aim of understanding the influence of the mechanical properties and composites on the release of the hydrophilic drug 5-fluorouracil (5-FU). The release was studied at acidic and neutral conditions to assess the pH effect and simulate the acidic pH conditions of tumor microenvironment and the slightly alkaline pH of physiological media, respectively.^58,59^ As displayed in Figure 5, the pH = 6 profiles displayed a faster release rate in the first 24 h, followed by a slower phase, which is in line with other chitosan/alginate gels for delivery of 5-FU.^17,18,60,61^ Besides, the drug was not completely released after 3 days, highlighting the role of the gel matrix as a diffusion barrier to 5-FU release.

**Figure 5:**
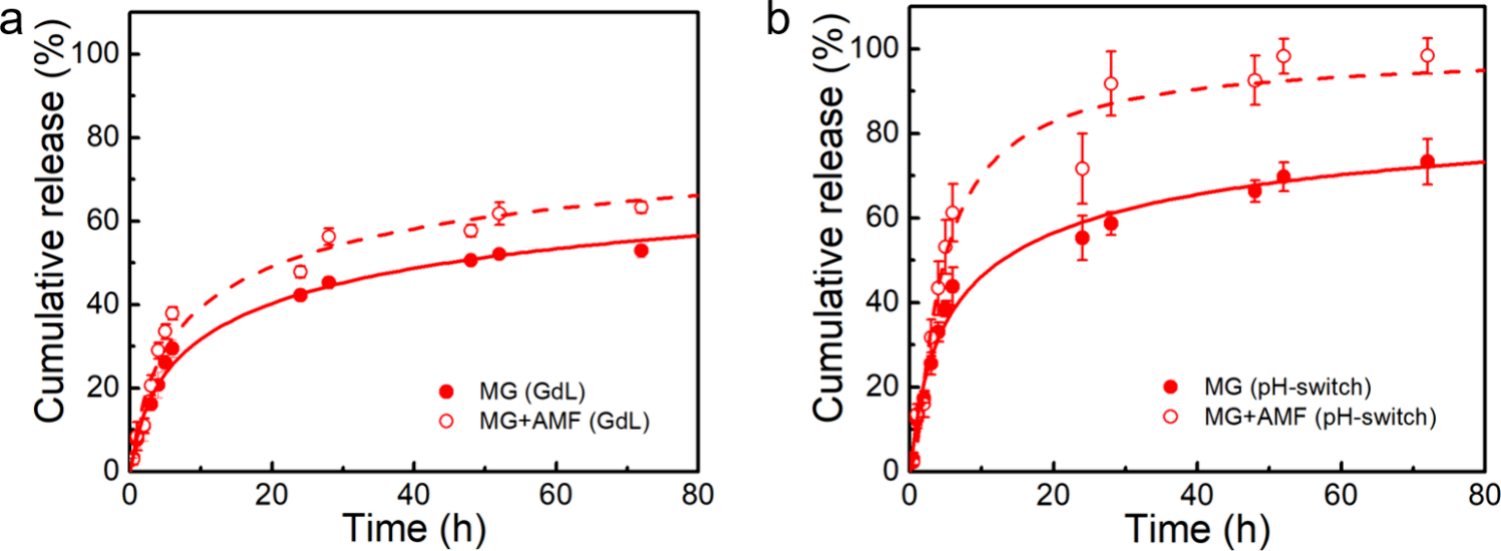
Cumulative 5-fluorouracil release at pH = 6 from magnetic gels (MG) prepared by the (**a**) GdL and (**b**) pH-switch methods. Active release was triggered with AMF at 617 kHz and 9.55 kA/m. The release profiles were fitted to the Gompertz model over 72 h.

Notably, the 5-FU release was enhanced in lower pH medium, achieving ∼80% after 3 days compared to ∼60% in neutral medium (Figure S15). This effect has been described for other chitosan-based gels^18,62^ and could be associated with the larger solubility of chitosan and alginate for pH < ∼6.5 and pH > ∼3.5, respectively.^2^ Besides, polyelectrolyte complexes of chitosan and alginate are reported to display a larger swelling between pH = 3 and 7,^63,64^ which consequently could enhance the drug release within this pH range.^65^

Despite the different rheological profiles, blending both gels with magnetic nanoparticles hampered the drug release, which could result from the nanoparticles working as cross-linkers or fillers.^50^ Regarding the former, as the magnetic gels displayed a similar or a slight decrease of the storage modulus compared to the hydrogels, the nanoparticles either did not affect the gel matrix or induced a decrease of the cross-linking density. In this sense, as also suggested from the TEM and SEM images, the nanoparticles may work as fillers that occupy the mesh water pockets and thus hamper the drug diffusion. Moreover, in line with the rheological properties, a loss of cross-linking density in magnetic gels prepared by pH-switch is also inferred from the drug release assays, as suggested by the larger drug release compared to the GdL-triggered magnetic gels.

The drug release was also carried out under exposure to an alternating magnetic field (AMF) to assess the influence of magnetic hyperthermia. The synergistic effect of magnetic hyperthermia and drug delivery is anticipated to enhance the therapeutic efficacy.^12,13^ In general, the results showed that exposure to AMF led to a slight enhancement of the release in the initial 24 h and also after each exposure during the slower phase. However, a stronger effect was obtained in the faster release phase, mainly in the pH-switch gels, which could result from the lower cross-linking density. Hence, having in mind the need to maintain drugs with therapeutic concentrations in the target site as well as to tune the release rate and duration, the developed magnetic gels hold promise for the delivery of 5-fluorouracil.

Further insight into the results was provided by the fitting of mathematical models (Tables S6 and S7), including the Korsmeyer–Peppas and Gompertz models.^66^ The latter was employed for comparison of the release kinetics, providing a good fitting of the release profiles. The former can be applied to different system geometries under perfect sink conditions, which also adequately described the results. Importantly, it confirmed that the nanoparticles hindered the release rate, while it was enhanced upon exposure of the magnetic gels to magnetic hyperthermia.

The parameter *n* of the Korsmeyer–Peppas model is related with the diffusion mechanism, which can be determined by fitting 60% of the drug release.^67^ In general, the initial release profiles displayed *n* values ∼ 0.89, suggesting that the release is primarily controlled by an anomalous transport mechanism (polymer relaxation and swelling), rather than diffusion, or a combination of both mechanisms. The anomalous transport mechanism was also observed for other chitosan/alginate gels,^18,60,61,65^ but a diffusion-mediated release is commonly reported due to fitting of the entire drug release profile. Importantly, the magnetic hyperthermia led to a faster drug diffusion as described for other magnetic gels,^13,50,68^ as well as a change to a release mechanism driven by polymer relaxation, further highlighting the influence of hyperthermia on both the drug release kinetics and mechanism. A nearly zero-order release kinetics can provide a constant drug release rate, enabling local steady therapeutic drug levels over time under magnetic hyperthermia. In this sense, the synergistic effect of constant local drug concentration, increased temperature, and enhanced drug release in acidic conditions under magnetic hyperthermia is anticipated to result in an improved therapeutic efficacy.

## 3 Conclusion

In this work, chitosan/alginate polyelectrolyte gels blended with magnetic nanoparticles were developed to achieve improved tunability of mechanical properties and sustained release of hydrophilic drugs. Gelation could be induced through slow (glucono-δ-lactone) and fast (phosphate buffer) pH decreases, and the influence of self-assembly conditions was studied. The polymer concentration, final pH, and kinetics of pH decrease provided control over the mechanical properties, enabling the formation of strong and highly deformable hydrogels. Notably, the kinetics of pH decrease influenced the gels’ rheological behavior, highlighting the interplay between self-assembly dynamics and mechanical properties. In parallel, superparamagnetic manganese ferrite nanoparticles with high saturation magnetization were synthesized via amino acid-assisted oxidative hydrothermal synthesis. The nanoparticles were blended in the chitosan/alginate gels at high concentrations without strongly compromising the mechanical properties of the hydrogels. The resulting magnetic hydrogels displayed high heating efficiency (ILP > 3 nHm^2^/kg) and improved sustained release of a hydrophilic drug model, 5-fluorouracil. The release was larger under acidic conditions and could be further enhanced through magnetic hyperthermia, demonstrating the synergistic potential of combining the magnetic and pH-responsive capability of chitosan/alginate magnetic gels. In this way, the results present polyelectrolyte chitosan/alginate magnetic gels as a versatile material with tunable mechanical properties, high heating efficiency, and sustained release capability, in which the release is enhanced through the combined effect of acidic pH and magnetic hyperthermia.

Considering that the biocompatibility and biodegradability of chitosan/alginate gels align with the growing demand for sustainable biomedical materials, the developed self-assembled pH-triggered chitosan/alginate-based magnetic gels are anticipated to pave the way for the development of natural polymer-based gel formulations for therapeutic applications, aiming for personalized therapy as a future perspective. Following these lines, future developments may include assessing long-term stability, efficacy, and safety of the gels *in vivo*, developing cytocompatible and biosafe magnetic particles with superior magnetic properties, and assessing the performance for delivery of other therapeutic agents. Addressing these challenges may contribute to the development of chitosan/alginate-based magnetic gels for a range of applications, including drug delivery (e.g., cancer therapy and wound healing). The combination of biologically active natural polymers with remote and spatiotemporal control of drug release and magnetic hyperthermia may minimize side effects, improve safety, and synergistically enhance the therapeutic outcome of the drug delivery system. In this way, chitosan/alginate-based magnetic gels are positioned as promising drug delivery platforms that could reduce the frequency of drug administration.

## 4 Experimental Section/Methods

### Chemicals

Trisodium citrate dehydrate, sodium hydroxide (NaOH), iron­(II) sulfate heptahydrate (FeSO_4_·7H_2_O), manganese­(II) sulfate monohydrate (MnSO_4_·H_2_O), sodium bicarbonate (NaHCO_3_), chitosan (448869, low molecular weight, 150–250 kDa, 75–85% deacetylation), alginate (180947, 120–190 kDa), l-glutamic acid (Glu), l-cysteine (Cys), l-glycine (Gly), l-phenylalanine (Phe), l-alanine (Ala), and l-asparagine (Asn) were purchased from Sigma-Aldrich. Milli-Q (MQ) grade water was used for all of the preparations.

### Characterization Techniques

UV/visible absorption spectra were recorded by using a Shimadzu UV-3600 Plus UV–vis–NIR spectrophotometer (Shimadzu Corporation, Kyoto, Japan). X-ray diffraction (XRD) analyses were conducted with a conventional PANalytical X’Pert PRO diffractometer (Malvern Panalytical Ltd., Malvern, UK), utilizing Cu K_α_ radiation, in a Bragg–Brentano configuration at the Electron Microscopy Unit, University of Trás-os-Montes and Alto Douro (UTAD), Vila Real, Portugal. Raman spectroscopy measurements were performed at room temperature with a Renishaw inVia Reflex Raman confocal microscope system (Wotton-under-Edge, Stroud, UK), equipped with a high-resolution grating of 1200 grooves mm^–1^. The excitation line, 785 nm, of a NIR diode laser was focused onto the sample by a 50× objective with a numerical aperture (NA) value of 0.40 in a backscattering geometry. The spectra were acquired with a measured power of about 650 μW on the sample, with a spectral acquisition time of 120 s over one accumulation and the range 100–1000 cm^–1^. The average hydrodynamic diameter and zeta potential of the nanoparticles (*n* = 3 independent runs) were measured in 10 mM phosphate buffer pH 6 and 7.4 at 0.01 mg/mL in Litesizer 500 dynamic light scattering (DLS) equipment from Anton Paar (Anton Paar GmbH, Graz, Austria), using a semiconductor laser diode of 40 mW and λ = 658 nm, a backscatter angle of 175°, and a controlled temperature of 25 °C. For the gels, Fourier transform infrared spectroscopy (FTIR) spectra were measured using a Nicolet 6700 spectrometer (CACTI, Vigo, Spain) with an attenuated total reflectance (ATR) accessory in the range between 4000 and 400 cm^–1^, using 32 scans with a resolution of 4 cm^–1^. For the magnetic nanoparticles, FTIR was measured using a Jasco FT/lR-6X FTIR spectrometer with an ATR accessory in the range between 4000 and 400 cm^–1^, using 50 scans with a resolution of 4 cm^–1^. Inductively coupled plasma optical emission spectroscopy (ICP-OES) measurements were carried out on an ICP-OES iCAP PRO XP Duo. TEM images were captured using a high contrast JEOL JEM-1010, operating at 100 kV (CACTI, Vigo, Spain). Scanning transmission electron microscopy (STEM) images were recorded using a NanoSEM – FEI Nova 200, operating at 15 kV, coupled to an electron dispersive spectroscopic analyzer (EDS) and electron backscatter diffraction EDAX – Pegasus X4M analyzer and detection system (EBSD) at SEMAT/UM (Guimarães, Portugal). The processing of STEM and TEM images was performed using ImageJ software (National Institutes of Health, NIH, Bethesda, MD, USA), which consisted of enhancing local contrast and adjusting brightness followed by selection of the nanoparticles. Magnetic measurements were performed on an MPMS3 SQUID magnetometer (Quantum Design Inc., San Diego, CA, USA). The field-dependent magnetization (hysteresis cycles) of the samples was measured in the large field range (up to *H* = 5570.42 kA/m) for each sample. In all the cases at 300 K, given the room temperature applications they are designed for, a specific magnetic field correction for the trapped flux in the superconducting coil was conducted, achieving an accuracy of residual less than 0.16 kA/m.^69^

### Synthesis of Manganese-Doped Ferrite Nanoparticles

The magnetic nanoparticles were prepared by oxidative hydrothermal synthesis.^15^ Briefly, the metal salts FeSO_4_·7H_2_O (1.33 mmol) and MnSO_4_·H_2_O (0.66 mmol) were dissolved in 1 mL of ultrapure water, which was added drop by drop to a 19 mL aqueous solution of amino acid (1 mmol, AA = Glu, Cys, Gly, Phe, Ala, Asn) and NaOH (4 mmol) under vigorous agitation and open to air. The reaction was kept for 30 min at room temperature, and a black precipitate was formed. The solution was transferred to a 50 mL Teflon-lined autoclave and kept in an oven for 24 h at 160 °C. The obtained particles were washed through magnetic decantation with water/ethanol 1:1 and dried at 80 °C.

### Preparation of Chitosan/Alginate Gels

To prepare chitosan/alginate gels, a 2 wt % chitosan basic solution was initially prepared, as described elsewhere.^4^ Briefly, 0.5 g of chitosan was dissolved in 25 mL of 0.1 M acetic acid solution, followed by the addition of 1 g of sodium bicarbonate, under vigorous stirring. Sodium alginate was dissolved in ultrapure water for a concentration of 2 wt %. The sodium alginate and chitosan solutions were mixed under a vortex, and the gelation was triggered by the addition of GdL (GdL method). The ratio and concentration of both polymers were screened, as well as the concentration of GdL. To prepare chitosan/alginate gels through the pH-switch method, the chitosan and alginate were mixed under vortex, and 0.1 mM phosphate buffer pH = 7.4 was added for a final concentration of 50 mM. For the preparation of magnetic gels, the required content of nanoparticles taken from a stock aqueous solution was centrifuged, and the pellet was redispersed in the chitosan/alginate solution prior to the addition of GdL. The drug-loaded gels were prepared for 0.2 mg/mL of 5-FU and a total chitosan/alginate concentration of 1 wt %, which corresponds to 0.5 wt % of each polymer.

### Drug Release Assays

Chitosan/alginate gel samples (200 μL) loaded with 5-FU were prepared and left to stabilize overnight in microcentrifugal tubes. Then, above the gel, 10 mM phosphate buffer pH = 7.4 or 6.0 (1 mL) was added to keep the pH constant. Aliquots (200 μL) were taken and replaced with a fresh buffer. The concentration at each time point was followed by UV–visible spectroscopy.^70^ The applied HF-AMF (high-frequency alternating magnetic field) for triggered release was a magnetic field of 617 kHz and 9.55 kA/m. Exposure to an HF-AMF was initiated after 3 h of passive release for 30 min in the interval between each time point.

### Magnetic Hyperthermia Measurements

The heating performance of the nanoparticles was studied by magneto-caloric measurements using a hyperthermia system magneTherm (nanoTherics, Warrington, UK), working at *f* ≈ (161, 270, 381, 616) kHz and at the magnetic field *H* = (13.56, 12.76, 9.55) kA/m. For all experiments, the initial temperature was stabilized at 25 °C before the measurement. Next, the AC magnetic field was applied, and the temperature was recorded by using a thermocouple. The samples were tested for a concentration of 5 mg/mL. A phenomenological Box–Lucas equation was fitted to the temperature variation profile,^15^ and the initial slope of the temperature curves were determined, d*T*/d*t*, which is required to calculate the specific loss power (SLP):

1 $$SLP=\frac{m_{sample}c}{m_{NP}}\frac{dT}{dt}$$

where *m*_*sample*_ is the total mass of the sample (g), *c* is the specific heat capacity of the sample (approximated to *c*_*water*_ = 4.185 J/g/K), and *m*_*NP*_ is the total mass of nanoparticles in the sample (g).

## Supplementary Material



## Notes

FTIR, ICP-OES, magnetic properties, TEM images XRD, and magnetic heating profiles of magnetic nanoparticles; zeta potential, additional STEM images, FTIR spectra, rheological characterization, SEM images, heating performance, and drug release results of gels (PDF)

## ABBREVIATIONS

AA: amino acid
Ala: alanine
Asn: asparagine
CGC: critical gelation concentration
Cys: cysteine
DNA: DNA
FTIR: Fourier transform infrared spectroscopy
5-FU: 5-fluorouracil
GdL: glucono-δ-lactone
Glu: glutamate
Gly: glycine
ICP-OES: inductively coupled plasma optical emission spectroscopy
PEC: polyelectrolyte complex
Phe: phenylalanine
SQUID: superconducting quantum interference device
STEM: scanning transmission electron microscopy
XRD: X-ray diffraction
