# Supplementary material for: Chitosan/Alginate Polyelectrolyte Magnetic Gel Nanoarchitectonics with Tunable Mechanical Properties for Magnetic Hyperthermia and Sustained Release of 5‑Fluorouracil
Source: ACS Appl Mater Interfaces. 2025 Oct 28;17(45):61731–41. doi: 10.1021/acsami.5c15863 (PMC12616591; doi:10.1021/acsami.5c15863)
Supplement: Supplementary file 1 [file am5c15863_si_001.pdf]

Supporting Information

A chitosan/alginate polyelectrolyte magnetic gel nanoarchitectonics with tuneable mechanical properties for magnetic hyperthermia and sustained release of 5-fluorouracil

Sérgio R. S. Veloso,[a,b,c,*] *Margarita Vázquez-González,*[c,*] *Mariana O. Ribeiro,*[a] *Loic Hilliou,*[d] *Carlos O. Amorim,*[e] *Vítor S. Amaral,*[f] *Miguel A. Correa-Duarte,*[c,*] *Elisabete M. S. Castanheira,*[a,b,*]

[a] Physics Centre of Minho and Porto Universities (CF-UM-UP), University of Minho, Campus de Gualtar, 4710-057 Braga, Portugal.

[b] LaPMET Associate Laboratory, University of Minho, Campus de Gualtar, 4710-057 Braga, Portugal.

[c] Centro de Investigación en Nanomateriais e Biomedicina (CINBIO), Universidad de Vigo, 36310 Vigo, Spain

[d] Institute for Polymers and Composites, Department of Polymer Engineering, University of Minho, Campus de Azurém, Guimarães, 4800-058 Portugal

[e] Physics Department and i3N, University of Aveiro, Campus de Santiago, Aveiro 3810-193, Portugal

[f] Physics Department and CICECO, University of Aveiro, Campus de Santiago, Aveiro 3810-193, Portugal

*(S.R.S.V.) E-mail: sergiorafael.dasilva@uvigo.gal

*(M.V.-G.) E-mail: margarita.vazquez@uvigo.gal

*(E.M.S.C.) E-mail: ecoutinho@fisica.uminho.pt

*(M.A.C.-D) E-mail: macorrea@uvigo.gal

Supporting Information

**Table of Contents**	**Page No.**
Characterization of the magnetic nanoparticles	3
Characterization of the hydrogel	10
Characterization of the magnetic gel	19
Drug release assays	22

1. Characterization of the magnetic nanoparticles

1.1. FTIR

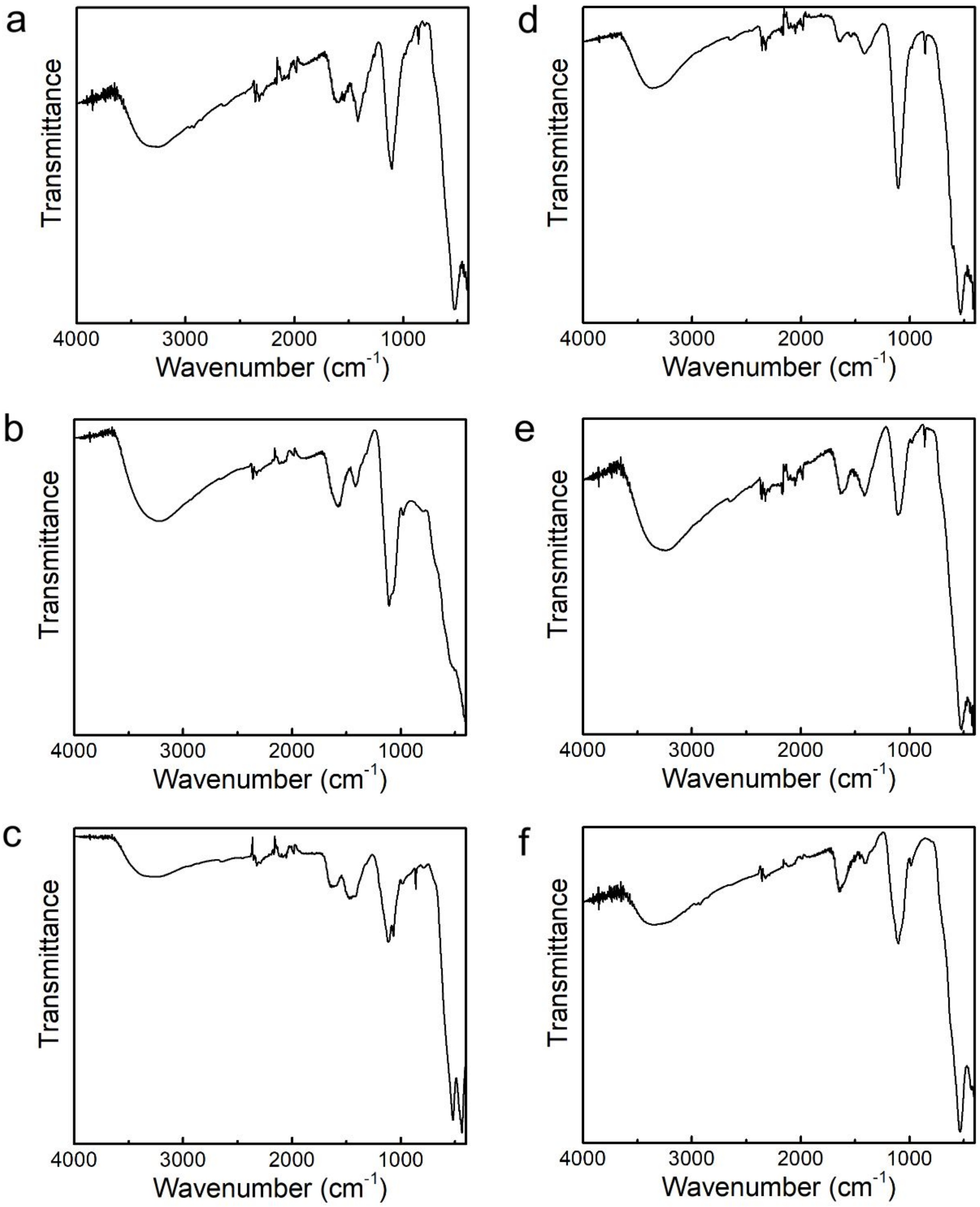

Figure S1. FTIR spectra of the manganese-doped ferrite nanoparticles prepared with (**a**) alanine, (**b**) asparagine, (**c**) cysteine, (**d**) glutamate, (**e**) glycine and (**f**) phenylalanine.

1.2. Metal composition

Table S1. ICP-OES determined composition of the manganese-doped ferrite nanoparticles synthesized with alanine, asparagine, cysteine, glutamate, glycine and phenylalanine.

	**S (mg/kg)**	**Fe (mg/kg)**	**Mn (mg/kg)**
**Alanine**	--	376098	147474
**Asparagine**	--	403894	97863
**Cysteine**	117688	477262	44869
**Glutamate**	--	272297	138110
**Glycine**	--	431885	163810
**Phenylalanine**	--	523945	88583

1.3. Magnetic properties

Table S2. Experimentally determined magnetic properties of manganese-doped ferrite nanoparticles measured at 300 K: saturation magnetization (M_S); remnant magnetization (M_R); squareness (M_R/M_S); coercivity (H_C). The saturation magnetization M_S was calculated using a law of approach to magnetic saturation (LAS), and was normalized for the mass of nanoparticle (NP) or iron (Fe) content.

AA	M_S (Am^2/kg_{NP})	M_S (Am^2/kg_{Fe})	H_C (kA/m)	M_R (Am^2/kg_{NP})	M_R/M_S

Alanine	64.5	171.5	2.5	3.3	0.05
Asparagine	17.8	43.9	0.5	0.2	0.01
Cysteine	19.9	41.7	21	5.8	0.29
Glutamate	54.8	201.4	2.7	3.3	0.06
Glycine	63.4	146.8	2.4	2.4	0.04
Phenylalanine	72.9	139.3	1.4	2.2	0.03

A law of approach to magnetic saturation (LAS) was used to calculated the saturation magnetization, M_s, which considers the contributions of magnetic anisotropy $\left(\frac{1}{H^2}\right)$, the high field paramagnetic and/or antiferromagnetic susceptibility(χH) and defects$\left(\frac{a}{H}\right)$,[1,2] as follows:

$$M(H) = M_s\left(1 - \frac{a}{H} - \frac{b}{H^2}\right) + \chi H \qquad \text{(S1)}$$

The $\frac{a}{H}$ and χH terms were found to be essential to properly describe the magnetization using LAS, while the $\frac{1}{H^2}$ displayed a negligible contribution.

1.4. Transmission electron microscopy

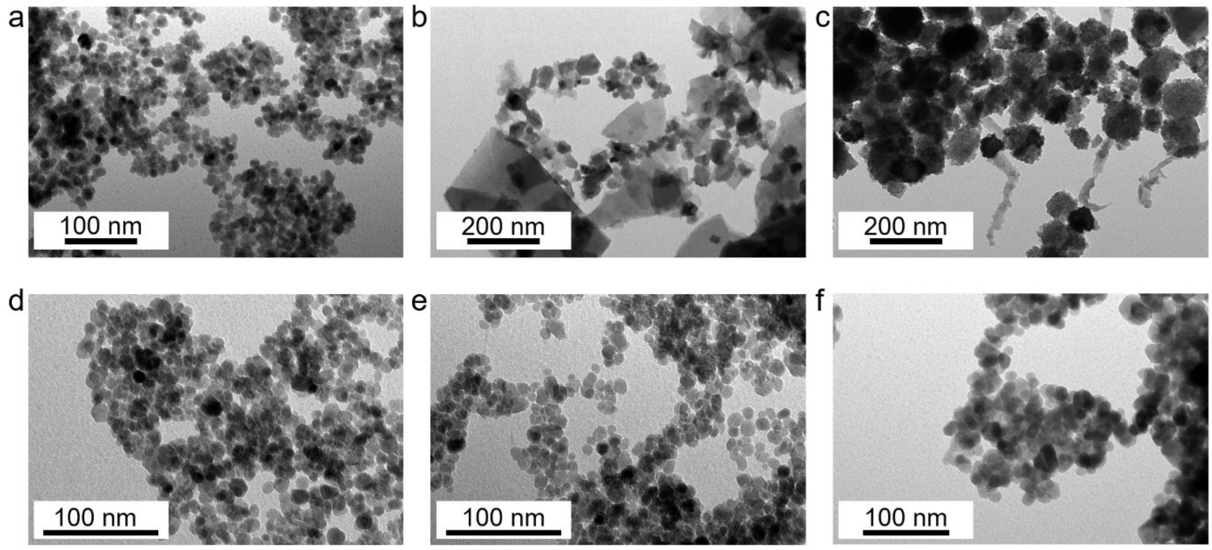

Figure S2. Electron microscopy of the manganese-doped ferrite nanoparticles prepared with (**a**) alanine, (**b**) asparagine, (**c**) cysteine, (**d**) glutamate, (**e**) glycine and (**f**) phenylalanine.

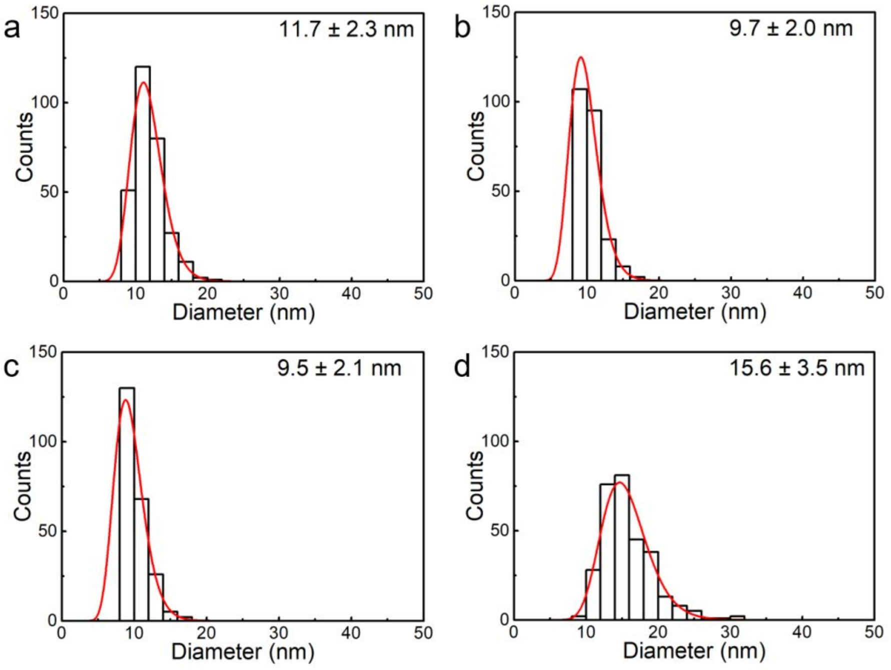

Figure S3. Size histograms of the manganese-doped ferrite nanoparticles prepared with (**a**) alanine, (**b**) glutamate, (**c**) glycine and (**d**) phenylalanine.

1.5. X-ray diffraction

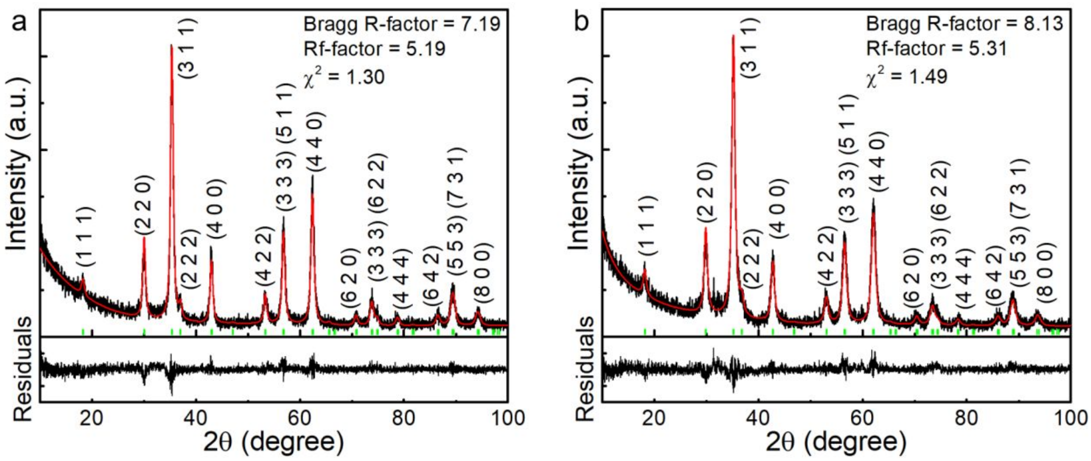

Figure S4. XRD diffraction pattern (black line) and fitted Rietveld refinement pattern (red line) of manganese-doped ferrites prepared with (**a**) phenylalanine and (**b**) alanine. The green bars highlight the Bragg diffraction pattern contributions from Fd3m (ferrite cubic phase) structures.

The Bragg's reflections characteristic of the Fd3m space group confirmed the cubic spinel structure, and the Miller indices of the diffraction peaks are included in **Figure S4**. The refinement resulted in a good quality fitting, as indicated from the parameters Rf and χ^2. An average crystallite size of 11.4 nm (a = 8.415(9) Å) and 8.4 nm (a = 8.455(9) Å) was obtained for Phe- and Ala-functionalized nanoparticles, respectively, which is smaller than the obtained particle size from TEM.

1.6. Magnetic hyperthermia

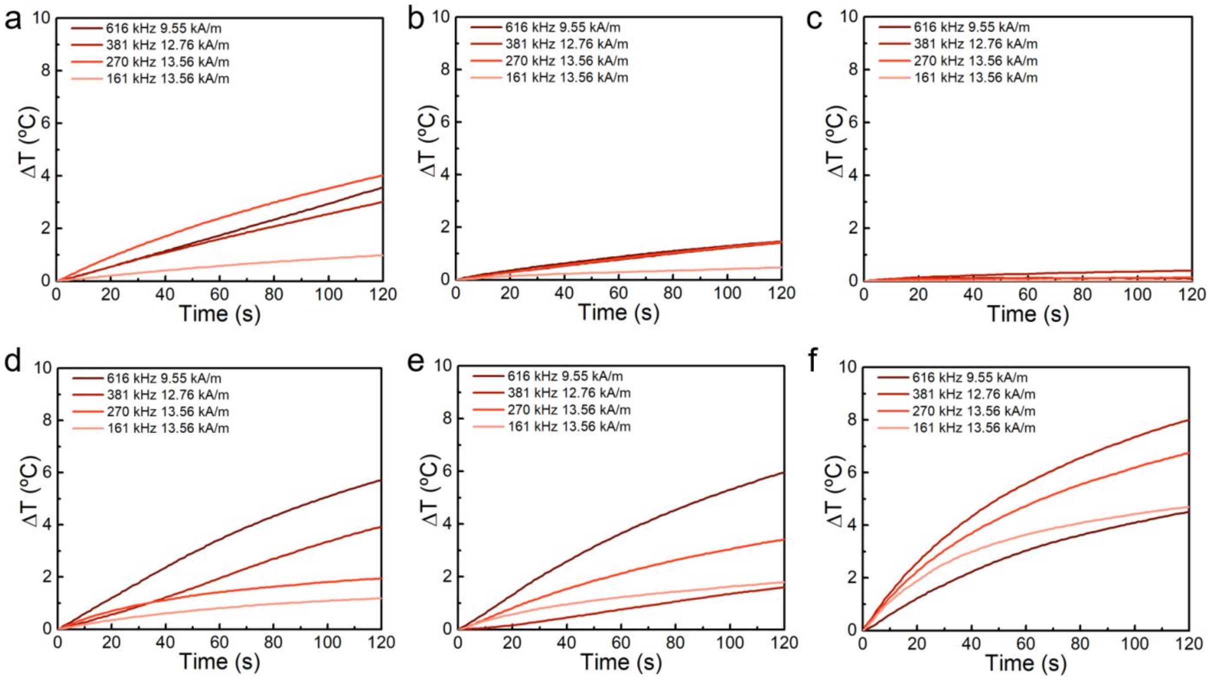

Figure S5. Temperature variation of manganese-doped ferrite nanoparticles prepared with (**a**) alanine, (**b**) asparagine, (**c**) cysteine, (**d**) glutamate, (**e**) glycine and (**f**) phenylalanine, under different alternating magnetic field conditions.

Table S3. Experimentally determined intrinsic loss power (nHm^2/kg) of the amino acid (AA)-prepared manganese-doped ferrite nanoparticles under alternating magnetic fields of variable intensity and frequency.

AA	9.55 kA/m 616 kHz	12.76 kA/m 381 kHz	13.56 kA/m 270 kHz	13.56 kA/m 161 kHz
Alanine	0.170	0.653	0.478	0.321
Asparagine	0.102	0.202	0.238	0.516
Cysteine	0.004	0.080	0.130	0.326
Glutamate	0.290	0.536	0.559	1.899
Glycine	0.473	0.604	0.225	2.087
Phenylalanine	1.723	1.689	2.436	1.967

2. Characterization of the hydrogel

2.1. pH and zeta potential

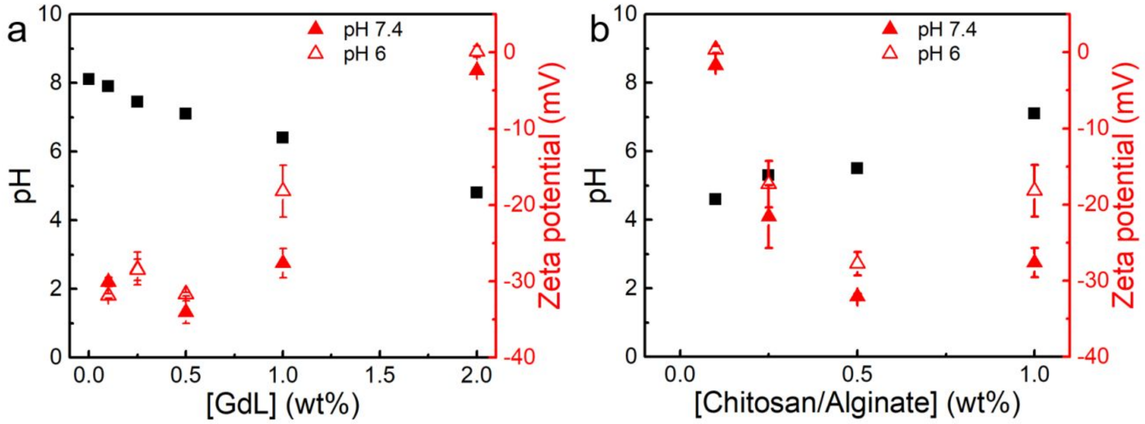

Figure S6. Dependence of pH and zeta potential on (**a**) concentration of glucono-δ-lactone (GdL) for a chitosan/alginate gel at 1 wt%, and (**b**) total polymer content for 1 wt% GdL. The gels were diluted 10× with phosphate buffer pH 6 or 7.4 prior to measurement of the zeta potential. The results demonstrate that the concentration (and ratio) of both polymer and GdL affect the final pH and zeta potential of the polyelectrolyte complex.

2.2. Gels morphology

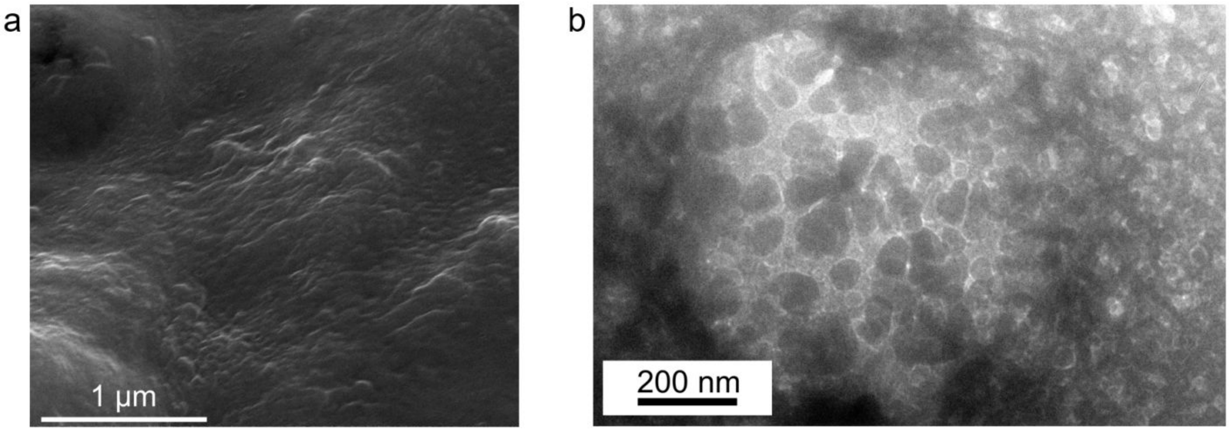

Figure S7. (**a**) SEM and (**b**) TEM images of the chitosan/alginate gels. Nanopores smaller than 200 nm are observed in TEM image, while the SEM image reveals a rough surface.

2.3. FTIR spectra

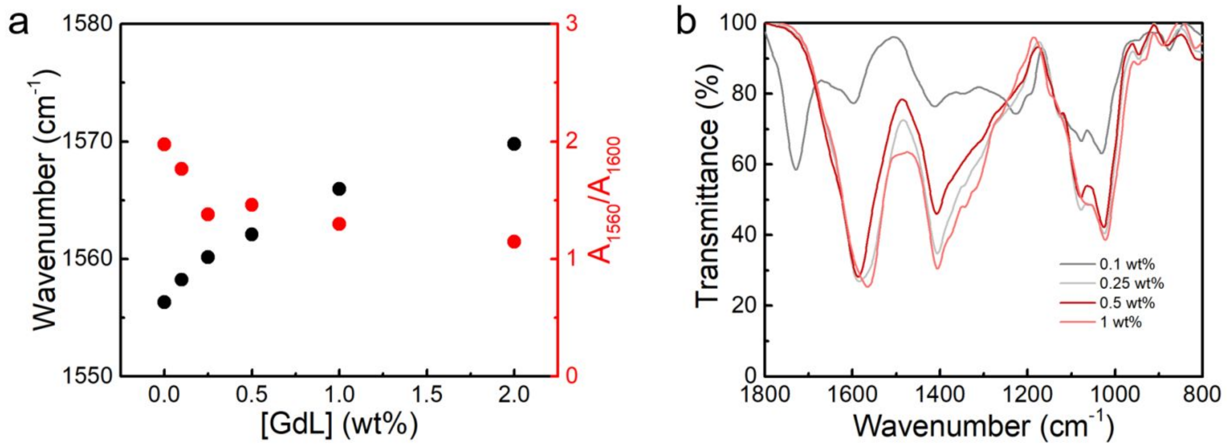

Figure S8. (**a**) Dependence of the maximum wavenumber of the broad band near 1555 cm^{-1} on the concentration of GdL for a 1 wt% gel, and the associated ratio of the absorbance at 1560 cm^{-1} and 1580 cm^{-1}. (**b**) FTIR spectra of chitosan/alginate polyelectrolyte solutions prepared at 1 wt% GdL and variable amount of chitosan. The incremental content of GdL is observed to induce a blue shift of the band near 1555 cm^{-1} at 0 wt% GdL.

2.4. Rheological assays

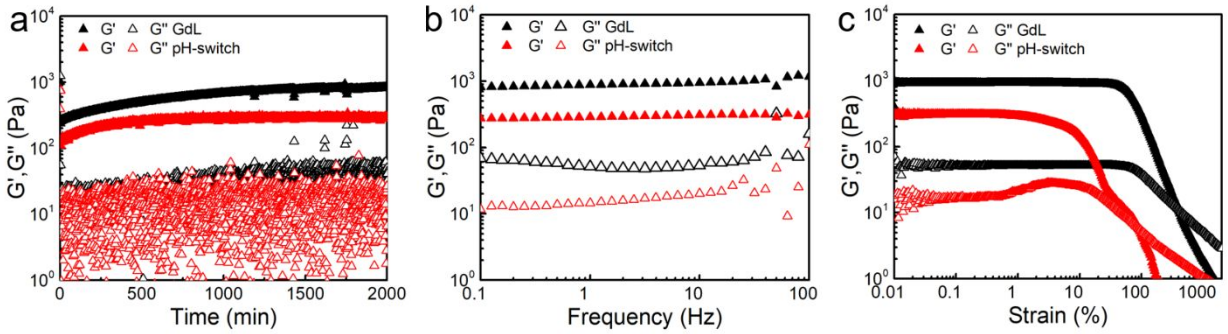

Figure S9. Dependence of the shear storage G’ (filled symbols) and loss G’’ (empty symbols) modulus of chitosan/alginate gels triggered by GdL or pH=7.4 phosphate buffer (pH-switch) during (**a**) the gelation process, (**b**) frequency and (**c**) strain sweeps.

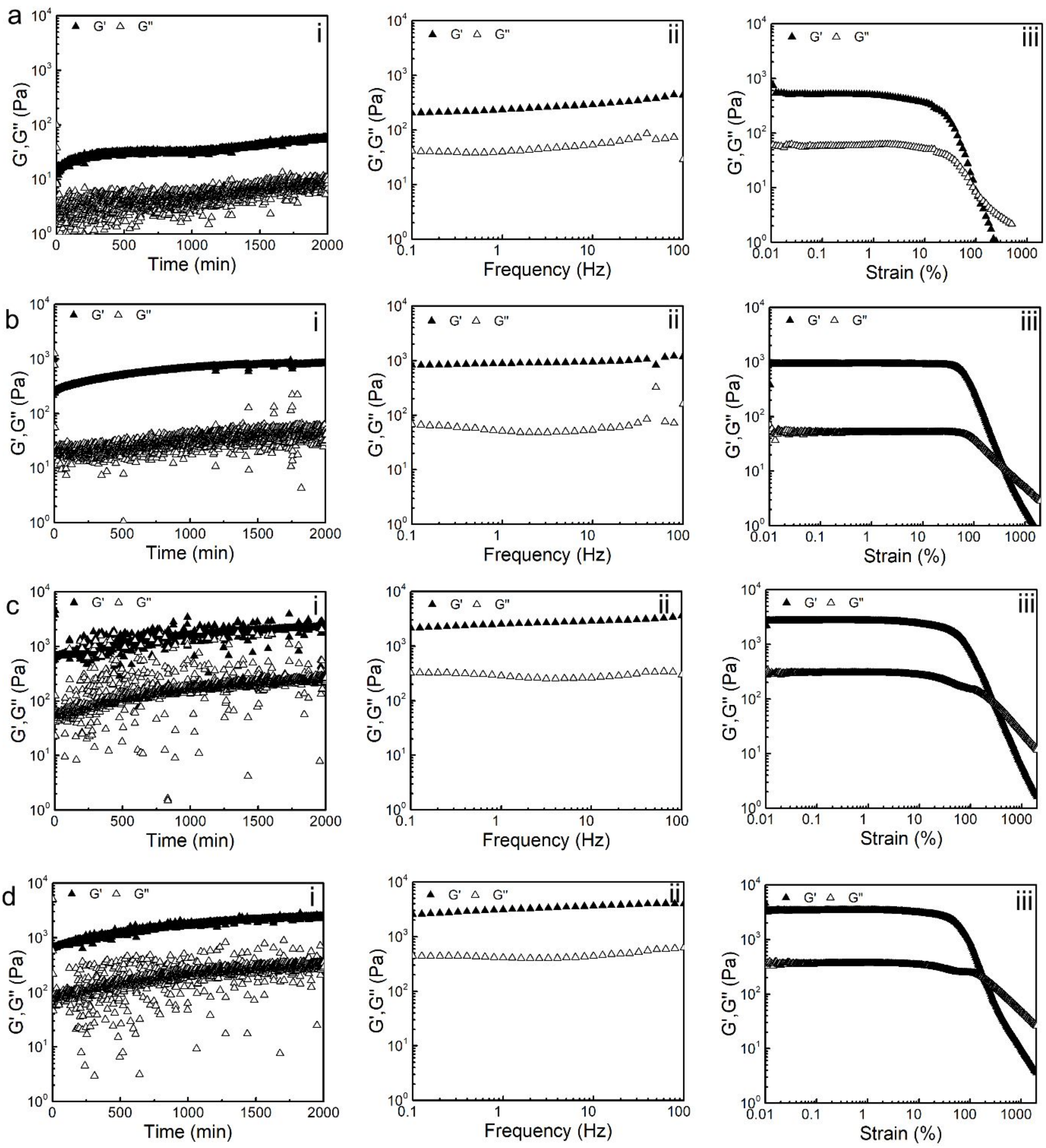

Figure S10. Dependence of the shear storage G' (filled symbols) and loss G'' (empty symbols) modulus of chitosan/alginate gels triggered by GdL (1 wt%) at a total polymer concentration of (**a**) 0.5 wt%, (**b**) 1 wt%, (**c**) 1.5 wt%, and (**d**) 2 wt% during (**i**) the gelation process, (**ii**) frequency and (**iii**) strain sweeps.

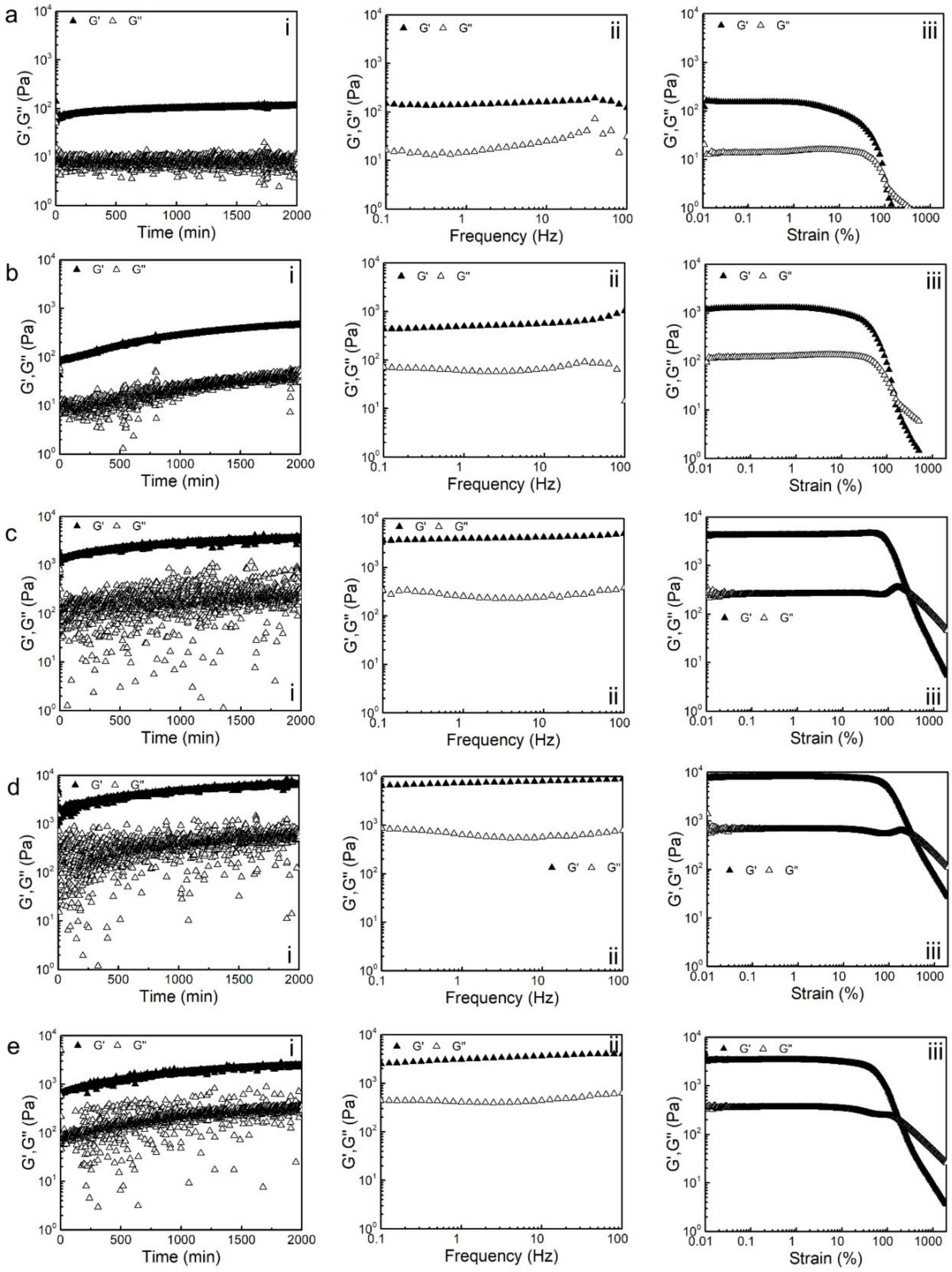

Figure S11. Dependence of the shear storage G' (filled symbols) and loss G'' (empty symbols) modulus of chitosan/alginate gels (2 wt%) triggered by GdL (**a**) 0.05 wt%, (**b**) 0.3 wt%, (**c**)

0.5 wt%, (**d**) 0.8 wt%, and (**e**) 1 wt% during (**i**) the gelation process, (**ii**) frequency and (**iii**) strain sweeps.

Table S4. List of chitosan-based gels preparation parameters, mechanical properties, and application. The polymer concentration employed in the rheological assays [Gel] are included. The approximate (or range) limit strain of the linear viscoelastic regime (LVR), critical strain (γ), storage (G′), and loss (G″) moduli are indicated unless the values are detailed in the respective manuscript.

Gel	Composite	Method	pH	[Gel] (wt%)	G’	G’’	LVR (%)	γ (%)	Application	Ref.
Chitosan/alginate (1:1)	$MnFe_2O_4$@Phe	GdL	7.1	1	750	57	3	420	Drug delivery	This work
Chitosan/alginate (1:1)	$MnFe_2O_4$@Phe	Phosphate buffer pH=7.4	7.4	1	345	16	1	30	Drug delivery	This work
Chitosan/alginate (1:10)	-	GdL	4.5	-	~60	~10	1	<100	-	[3]
Chitosan/alginate	-	HCl	7	2	<10	<10	-	-		[4]

(1:2)										
Chitosan/alginate (1:1)	-	HCl	7	2	~50	~10	-	-	Citral encapsulation	
Chitosan/alginate (2:1)	-	HCl	7	2	~90	~40	-	-		
Chitosan/collagen (1:1)	-	-	3.2	1.5	150	~10	-	13.1	Tissue engineering	5
	-	Fructose	3.2	1.5	219	~20	-	20.7	Tissue engineering	5
	-	Genipin	3.2	1.5	478	~30	-	≥100	Tissue engineering	5
	-	Transglutaminase	3.2	1.5	260	~20	-	13.2	Tissue engineering	5

	-	EDC/NHS	3.2	1.5	529	~40	-	29.3	Tissue engineering	5
Chitosan/collagen (1:1)	-	Genipin	-	2.6	10	1	<10	-	Diabetic wound healing	6
Chitosan	-	Michael addition reaction	5–6	0.5	975	452%	-	-	-	7
Chitosan/methacrylate gelatin (1:1)	-	Thiol-Michael addition reaction	-	2.5	~300	~50	40	~800	Wound healing	8
Chitosan	Polydiallyldimethylammonium chloride-coated γ-Fe_2O_3	Glyoxal	4.4	1.3	<100	<10	-	-	-	9

Chitosan/dialdehyde sodium alginate	Fe_3O_4	Aldehyde	-	-	1800-8900	100-1000	-	-	Drug delivery	[10]
Chitosan/carboxymethyl chitosan	Fe_3O_4-loaded chitosan microspheres	NaCl	-	-	400	~80	-	-	Drug delivery	[11]
Chitosan	Polyglucose sorbitol carboxymethylether (PSC)-coated γ-Fe_2O_3	β-glycerophosphate	-	-	~200	~10	-	-	Myocardial infarction treatment	[12]

3. Characterization of the magnetic gel

3.1. Morphology

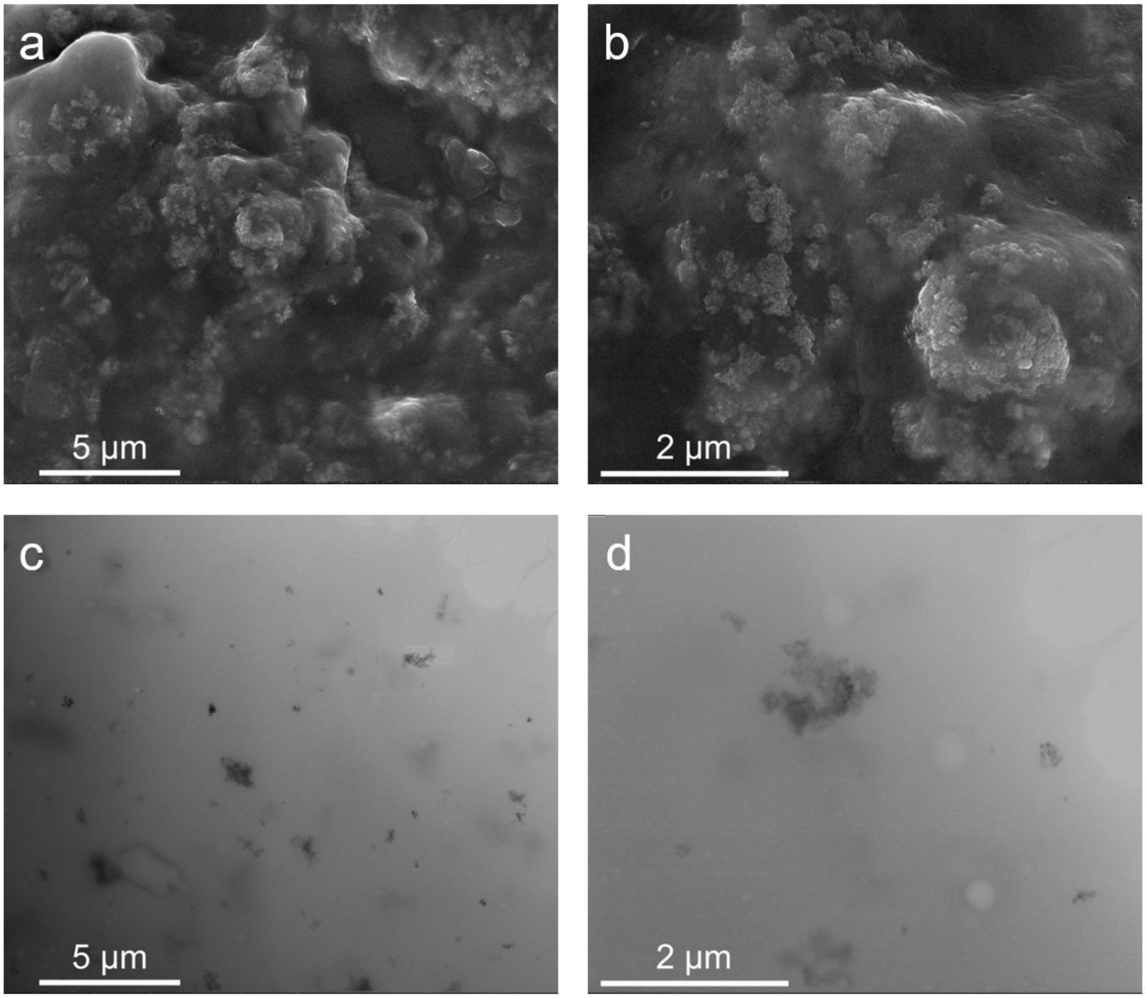

Figure S12. (**a,b**) SEM and (**c,d**) TEM images of the magnetic gels at different magnification.

3.2. Rheological properties

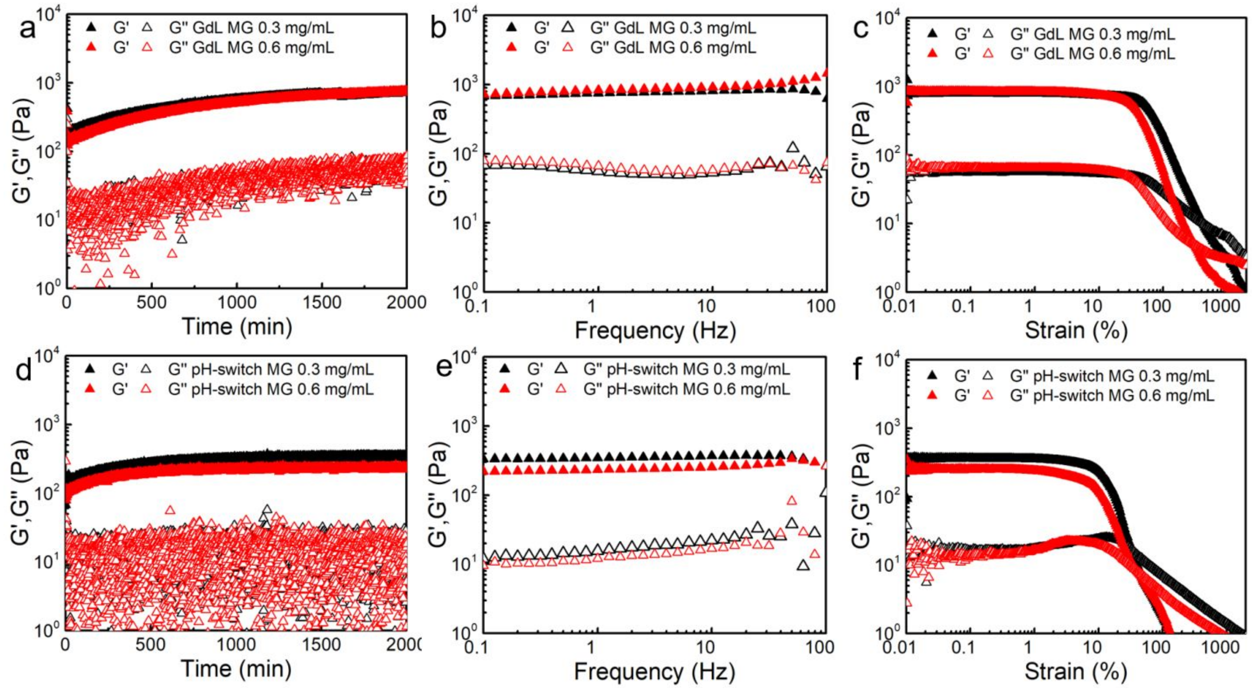

Figure S13. Dependence of the shear storage G' (filled symbols) and loss G'' (empty symbols) modulus of chitosan/alginate gels (1 wt%) triggered by (**a-c**) GdL and (**d-f**) phosphate buffer pH=7.4 during (**a**,**d**) the gelation process, (**b**,**e**) frequency and (**c**,**f**) strain sweeps.

3.3. Heating performance under an alternating magnetic field

Table S5. Experimentally determined intrinsic loss power (nHm^2/kg) of the magnetic gels containing 0.3 or 5 mg/mL of magnetic nanoparticles under alternating magnetic fields of variable intensity and frequency.

Nanoparticle concentration (mg/mL)	9.55 kA/m 616 kHz	12.76 kA/m 381 kHz	13.56 kA/m 270 kHz	13.56 kA/m 161 kHz
0.3	3.95	3.23	3.68	5.56
5	0.51	0.44	0.44	0.41

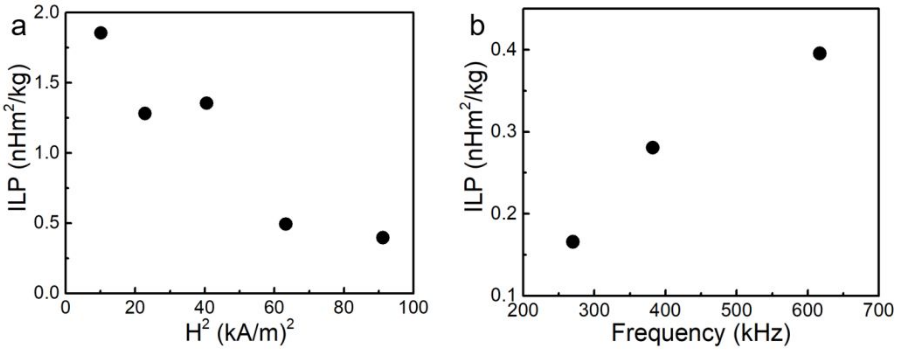

Figure S14. Dependence of the intrinsic loss power of magnetic gels with 5 mg/mL of magnetic nanoparticles on the (**a**) amplitude at 616 kHz and (**b**) frequency at 9.55 kA/m of the magnetic field.

4. Drug release assays

Table S6. Coefficients of determination (R^2) of several fitted models obtained for 5-fluorouracil release profiles in hydrogels (HG) and magnetic gels (MG) prepared by GdL and pH-switch methods. The mathematical models were fitted to the 72 h release profiles.

Method	pH	Sample	First-order	Hixson-Crowell	Higuchi	Korsmeyer-Peppas	Gompertz
pH-switch	6	HG	0.87	0.42	0.77	0.92	0.99
		MG	0.80	0.28	0.78	0.92	0.98
		MG+AMF	0.94	0.61	0.79	0.89	0.97
	7.4	HG	0.78	0.23	0.79	0.89	0.98
		MG	0.80	0.27	0.83	0.94	0.99
		MG+AMF	0.78	0.27	0.78	0.90	0.95
GdL	6	HG	0.92	0.55	0.86	0.95	0.99
		MG	0.81	0.33	0.85	0.93	0.99

	MG+AMF	0.78	0.28	0.79	0.90	0.97
	HG	0.83	0.35	0.87	0.95	0.99
7.4	MG	0.76	0.01	0.78	0.95	0.99
	MG+AMF	0.78	0.25	0.80	0.91	0.97

The Gompertz and Korsmeyer-Peppas models are, respectively, described according to the equations:

$$X_t = X_{max} e^{-ae^{b \log_{10} t}} \tag{S2}$$

$$\frac{M_t}{M_\infty} = K_s t^n \tag{S3}$$

in which $\frac{M_t}{M_\infty}$ is the fraction of drug released at time t, and K_s is the rate constant. For a cylindrical geometry, when $n < 0.45$, the release mechanism is diffusion-controlled (Fickian diffusion), $0.45 < n < 0.89$ is an anomalous transport (comparable diffusion and swelling rates), and $n \geq 0.89$ indicates that the release is mainly driven by swelling or relaxation of network chains (case-II transport).[13,14] The X_t and X_{max} are the dissolved drug fractions at time t and its maximum, a is a shape parameter and b is the dissolution rate per unit of time.[15]

Table S7. Release coefficients of the Korsmeyer-Peppas and Gompertz models obtained for 5-fluorouracil release profiles in hydrogels (HG) and magnetic gels (MG) prepared by GdL and pH-switch methods. The Korsmeyer-Peppas model was fitted to the initial 60% of the drug release profile. The parameter X_{max} of the Gompertz model was fixed at value 1.

			**Korsmeyer-Peppas**			**Gompertz**			
**Method**	**pH**	**Sample**	K_S (h^{-1})	n	R^2	X_{max}	a	b	R^2
pH-switch	6	HG	0.11	0.91	0.99	1.00	2.09	1.43	0.99
		MG	0.08	0.95	0.99	1.00	2.11	1.01	0.98
		MG+AMF	0.08	1.15	1.00	1.00	2.93	2.11	0.97
	7.4	HG	0.07	0.94	0.99	1.00	2.22	0.83	0.98
		MG	0.06	0.93	1.00	1.00	2.42	0.74	0.99
		MG+AMF	0.05	1.16	1.00	1.00	2.30	0.92	0.95

GdL	6	HG	0.11	0.82	1.00	1.00	2.31	1.42	0.99
		MG	0.05	0.98	1.00	1.00	2.50	0.78	0.99
		MG+AMF	0.06	1.07	1.00	1.00	2.31	0.91	0.97
	7.4	HG	0.06	0.91	1.00	1.00	2.52	0.75	0.99
		MG	0.06	0.75	1.00	1.00	2.46	0.52	0.99
		MG+AMF	0.05	1.07	1.00	1.00	2.37	0.81	0.97

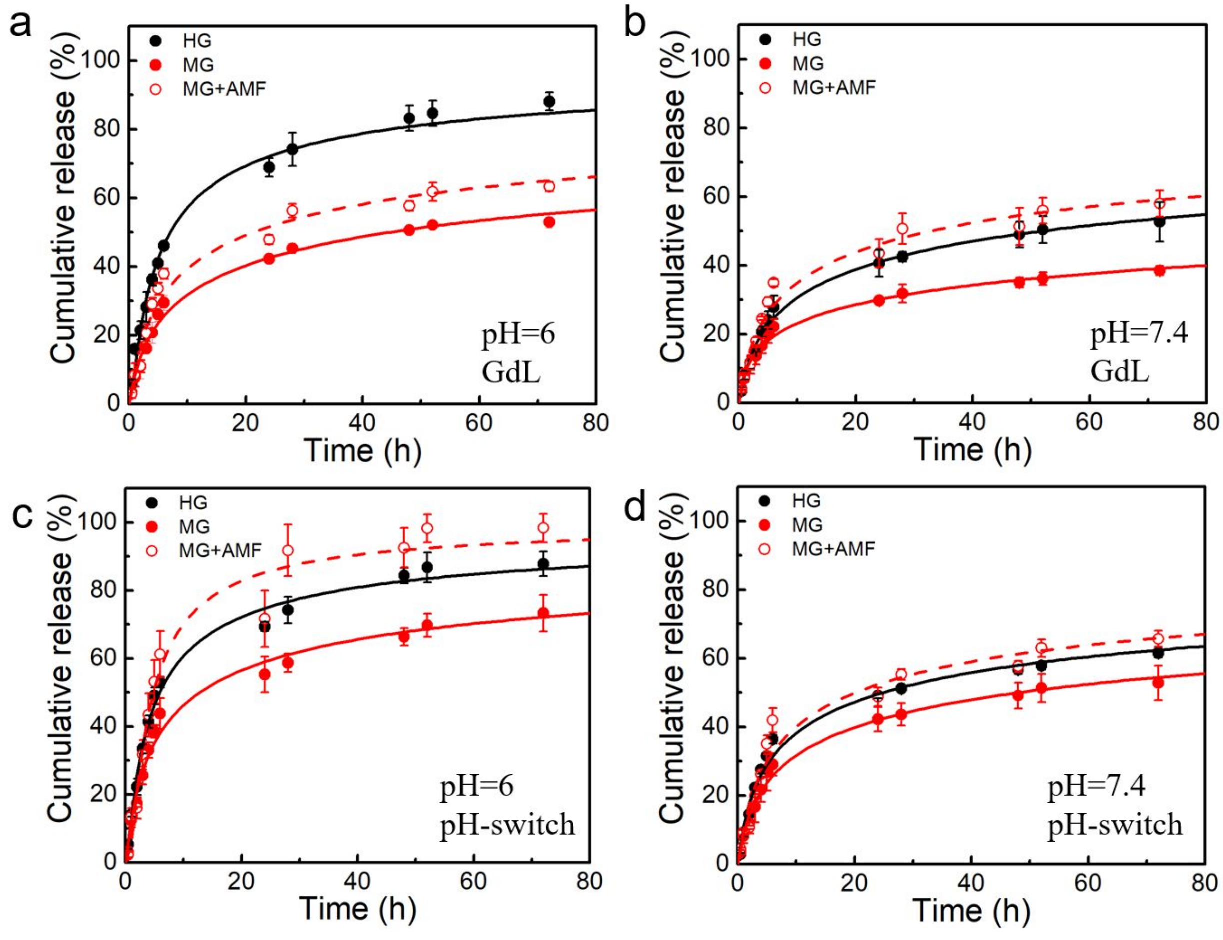

Figure S15. Cumulative 5-fluorouracil release at (**a**,**c**) pH=6 and (**b**,**d**) pH=7.4 from hydrogels (HG) and magnetic gels (MG) prepared by (**a**,**b**) GdL and (**c**,**d**) pH-switch methods. Active release

was triggered with AMF 617 kHz and 9.55 kA/m. The release profiles were fitted to Gompertz model over the 72 h.

References

(1) Chikazumi, S. *Physics of Ferromagnetism*; Oxford University Press, 2009.

(2) Grössinger, R. A Critical Examination of the Law of Approach to Saturation. I. Fit Procedure. *Phys. Status Solidi* **1981**, *66* (2), 665–674. https://doi.org/10.1002/pssa.2210660231.

(3) Komoto, D.; Furuike, T.; Tamura, H. Preparation of Polyelectrolyte Complex Gel of Sodium Alginate with Chitosan Using Basic Solution of Chitosan. *Int. J. Biol. Macromol.* **2019**, *126*, 54–59. https://doi.org/10.1016/j.ijbiomac.2018.12.195.

(4) Afzal, S.; Maswal, M.; Dar, A. A. Rheological Behavior of PH Responsive Composite Hydrogels of Chitosan and Alginate: Characterization and Its Use in Encapsulation of

Citral. *Colloids Surfaces B Biointerfaces* **2018**, *169*, 99–106. https://doi.org/10.1016/j.colsurfb.2018.05.002.

(5) Sánchez-Cid, P.; Alonso-González, M.; Jiménez-Rosado, M.; Benhnia, M. R.-E.-I.; Ruiz-Mateos, E.; Ostos, F. J.; Romero, A.; Perez-Puyana, V. M. Effect of Different Crosslinking Agents on Hybrid Chitosan/Collagen Hydrogels for Potential Tissue Engineering Applications. *Int. J. Biol. Macromol.* **2024**, *263*, 129858. https://doi.org/10.1016/j.ijbiomac.2024.129858.

(6) Li, Z.; Qian, C.; Zheng, X.; Qi, X.; Bi, J.; Wang, H.; Cao, J. Collagen/Chitosan/Genipin Hydrogel Loaded with Phycocyanin Nanoparticles and ND-336 for Diabetic Wound Healing. *Int. J. Biol. Macromol.* **2024**, *266*, 131220. https://doi.org/10.1016/j.ijbiomac.2024.131220.

(7) Guaresti, O.; Basasoro, S.; González, K.; Eceiza, A.; Gabilondo, N. In Situ Cross–Linked Chitosan Hydrogels via Michael Addition Reaction Based on Water–Soluble Thiol–Maleimide Precursors. *Eur. Polym. J.* **2019**, *119*, 376–384. https://doi.org/10.1016/j.eurpolymj.2019.08.009.

(8) Wu, Q.; Wang, L.; Ding, P.; Deng, Y.; Okoro, O. V.; Shavandi, A.; Nie, L. Mercaptolated Chitosan/Methacrylate Gelatin Composite Hydrogel for Potential Wound Healing Applications. *Compos. Commun.* **2022**, *35*, 101344. https://doi.org/10.1016/j.coco.2022.101344.

(9) Ikhaddalene, S.; Zibouche, F.; Ponton, A.; Irekti, A.; Carn, F. Synthesis and Rheological Properties of Magnetic Chitosan Hydrogel. *Period. Polytech. Chem. Eng.* **2021**, *65* (3), 378–388. https://doi.org/10.3311/PPch.17148.

(10) Chen, L.; Deng, X.; Tian, L.; Xie, J.; Xiang, Y.; Liang, X.; Jiang, L.; Jiang, L. Preparation and Properties of Chitosan/Dialdehyde Sodium Alginate/Dopamine Magnetic Drug-Delivery Hydrogels. *Colloids Surfaces A Physicochem. Eng. Asp.* **2024**, *680*, 132739. https://doi.org/10.1016/j.colsurfa.2023.132739.

(11) Wang, Z.; Zhai, X.; Fan, M.; Tan, H.; Chen, Y. Thermal-Reversible and Self-Healing Hydrogel Containing Magnetic Microspheres Derived from Natural Polysaccharides for Drug Delivery. *Eur. Polym. J.* **2021**, *157*, 110644. https://doi.org/10.1016/j.eurpolymj.2021.110644.

(12) Bao, S.; Lu, Y.; Zhang, J.; Xue, L.; Zhang, Y.; Wang, P.; Zhang, F.; Gu, N.; Sun, J. Rapid Improvement of Heart Repair in Rats after Myocardial Infarction by Precise Magnetic Stimulation on the Vagus Nerve with an Injectable Magnetic Hydrogel. *Nanoscale* **2023**, *15* (7), 3532–3541. https://doi.org/10.1039/D2NR05073K.

(13) Marcos Luciano Bruschi. *Strategies to Modify the Drug Release from Pharmaceutical Systems*; 2015.

(14) Ritger, P. L.; Peppas, N. A. A Simple Equation for Description of Solute Release I. Fickian

and Non-Fickian Release from Non-Swellable Devices in the Form of Slabs, Spheres, Cylinders or Discs. *J. Control. Release* **1987**, *5* (1), 23–36. https://doi.org/10.1016/0168-3659(87)90034-4.

(15) Dash, S.; Murthy, P. N.; Nath, L.; Chowdhury, P. Kinetic Modeling on Drug Release from Controlled Drug Delivery Systems. *Acta Pol. Pharm. - Drug Res.* **2010**, *67* (3), 217–223.
